# Culture-free detection of bacteria from blood for rapid sepsis diagnosis

**DOI:** 10.1038/s41746-025-01948-w

**Published:** 2025-08-25

**Authors:** M. Henar Marino Miguélez, Mohammad Osaid, Erik Hallström, Kerem Kaya, Jimmy Larsson, Vinodh Kandavalli, Carolina Wählby, Johan Elf, Wouter van der Wijngaart

**Affiliations:** 1 Micro and Nanosystems, KTH Royal Institute of Technology, Stockholm, Sweden; 2 Molecular Systems Biology, Uppsala University, Uppsala, Sweden; 3 Department of Information Technology, Uppsala University, Uppsala, Sweden; 4 SciLifeLab, Uppsala University, Uppsala, Sweden

**Keywords:** Biological techniques, Diseases, Medical research, Microbiology

## Abstract

Approximately 50 million people suffer from sepsis yearly, and 13 million die from it. For every hour a patient with septic shock is untreated, their survival rate decreases by 8%. Therefore, rapid detection and antibiotic susceptibility profiling of bacterial agents in the blood of sepsis patients are crucial for determining appropriate treatment. Here, we introduce a method to isolate bacteria from whole blood with high separation efficiency through *Smart centrifugation*, followed by microfluidic trapping and subsequent detection using deep learning applied to microscopy images. We detected, within 2 h, *E. coli*, *K. pneumoniae*, or *E. faecalis* from spiked samples of healthy human donor blood at clinically relevant concentrations as low as 9, 7 and 32 colony-forming units per ml of blood, respectively. However, the detection of *S. aureus* remains a challenge. This rapid isolation and detection represents a significant advancement towards culture-free detection of bloodstream infections.

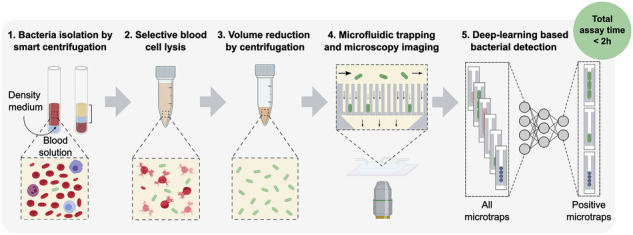

## Introduction

Sepsis is a severe medical condition characterized by a systemic host inflammatory response to an infection^1^. With an incidence of ~50 million cases annually,^2^ sepsis represents a significant healthcare burden. The mortality of diagnosed patients is around 26%^3^, and chances of survival drop dramatically if treatment is delayed^4^. An estimated 25–30% of sepsis cases involve bloodstream infections (BSI)^5,6^. Rapid detection and identification of pathogens in the patient’s blood is therefore important in order to initiate effective antibiotic treatment quickly.

The low microbial loads in the bloodstream of patients, as low as one to ten colony-forming units (CFUs) per ml of blood^7^, require large blood sample volumes, which is particularly challenging for pediatric patients^8^. Furthermore, to confirm the presence of bacteria in the blood and identify the species, several hours to days of culture are usually needed^9,10^. This delay is problematic considering the 8%-drop in the survival rate per hour of delayed treatment for patients suffering from septic shock^4,11^. The urgency of the condition, coupled with the latency of the available diagnosis methods, means that combination therapy with broad-range antibiotics is often prescribed as first-line therapy immediately, before the result of the diagnostic test has been obtained^12^. This praxis results in suboptimal treatment^13^, contributes to increased antibiotic resistance^14^, and has been shown to increase liver toxicity compared to targeted antibiotic monotherapy^15^.

The state-of-the-art methods for identifying bacteria from the blood of sepsis patients can be divided into^16,17^ genotypic (e.g., polymerase chain reaction), phenotypic (e.g., subcultures), or mass spectrometry methods (e.g., matrix-assisted laser desorption ionisation time-of-flight mass spectrometry (MALDI-TOF))^18^. While genotypic methods can identify bacteria at low concentrations without culturing the blood^18^, they do not provide susceptibility information^19^, limiting clinical impact. Phenotypic and proteomic approaches typically require a higher bacterial load, necessitating blood cultures and thus long processing times.

Traditional antibiotic susceptibility testing (AST) methods in the clinical setting rely on disk diffusion or broth microdilution, which can take several hours up to a day to yield results^20^. These methods rely on isolated cultures which require two previous culture steps, thus significantly increasing the total turnaround times to several days. New rapid EUCAST methodologies have significantly shortened the process, enabling testing directly from blood cultures, and providing results in as little as 4 to 8 h^21–23^. Moreover, in recent years, substantial progress has been made in developing novel AST methods that can be applied directly to positive blood cultures. For instance, systems like the PA-100 from SYSMEX has been shown to predict phenotypic resistance from positive blood cultures^24^. There are also 3D microwell-based chips that can perform AST using resazurin as a sensing probe^25^. Specific metabolic enzyme-based AST, such as fluorescence lifetime imaging microscopy (FILM), which measures metabolic perturbations after antibiotic exposure, can differentiate susceptible and resistant bacteria within 10–60 min^26^. In addition, there are pathogen-specific metabolic enzymes that serve as reliable AST biomarkers and can enable AST within 30 min^27–29^.

Further, the emergence of artificial intelligence (AI) systems has given rise to AI-powered technologies that use machine learning to interpret complex growth patterns, image-based phenotypes, or spectral data. AI algorithms embedded in platforms like Accelerate Pheno and dRAST analyze real-time bacterial responses, enhancing accuracy and reducing time to result to 4–7 h^30,31^. From a commercial perspective, companies like Q-linea, Gradientech, and QuantaMatrix offer integrated solutions capable of delivering AST results directly from positive blood culture samples within 4 to 6 h^32^. Molecular AST platforms, such as FilmArray (bioMérieux, Inc., Hazelwood, MO) and Verigene (Nanosphere, Northbrook, IL), can provide identification of several species and a narrow panel of antimicrobial resistance markers^22,33,34^. Despite these significant advances, the main bottleneck remains in the culture time required to obtain a positive blood culture, which can take several days for certain fastidious organisms^35^. Our approach focuses on solving this bottleneck.

Single-cell phenotypic methods, which bypass the requirement for blood culture, offer a quicker alternative but require that the bacteria be isolated from the excess of host blood cells^36–40^.

Isolation of bacteria from whole blood has been accomplished with inertial^41–43^ and elastoinertial microfluidics^44^, sedimentation velocity-based separation^45–48^, filtration^45,49^, chemical capture^50,51^, magnetic bead-based separation^50,52^, dielectrophoresis^53^, or acoustic separation^54^. However, most of these isolation methods suffer limited throughput and low blood cell rejection rates, or have only been demonstrated for bacteria concentrations of 1000 CFU/ml or above, thus necessitating time-consuming bacterial preculture.

This study aims to develop a rapid, high-throughput assay for isolating bacteria from whole blood with high efficiency and minimal dilution by trapping them in a microfluidic chip. In the chip, the bacteria are optically identified with a deep learning-based detection algorithm. The whole assay takes less than a few hours.

## Results

### Overall work-flow

**Fig. 1:**
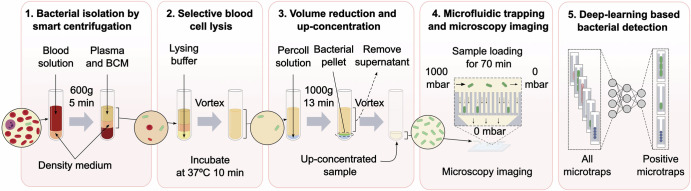
Workflow for bacterial detection from blood samples in five assay steps. **1** Isolation using smart centrifugation; **2** selective blood cell lysis; **3** volume reduction; **4** microfluidic trapping and microscopy imaging; and **5** deep-learning based bacterial detection. BCM is blood culture medium. Created in BioRender. Lab, E. (2025) https://BioRender.com/gyvhthfand in open-source software Inkscape.

Our assay concatenates five steps to isolate and detect bacteria from blood: smart centrifugation, selective blood cell lysis, volume reduction, microfluidic trapping combined with miscroscopy imaging, and deep-learning based detection of bacterial cells (Fig. 1).

To ensure reproducibility during the technical development, this study was conducted with healthy human EDTA donor blood, with all the blood parameters within the normal count. The blood was spiked with bacteria at concentrations, C, in the range 4–4000 CFU/ml. To facilitate development and quantification, we used a fluorescent lab *Escherichia coli* strain. The fluorescence facilitated the easy distinction between the *E. coli* test strain and potential contaminating bacteria, none of which were observed. Method evaluation was performed with clinical isolates of *Klebsiella pneumoniae*, *Enterococcus faecalis*, and *Staphylococcus aureus*, in their exponential growth phase, to resemble the clinical scenario^55^.

Details about materials and methods are provided under section “Methods and all measurement values” are tabled in SI section “Tabled experimental measurements and their results”.

### Smart centrifugation

The first assay step, which we call smart centrifugation, removes most of the blood cells while recovering most of the bacteria in the supernatant. This step is essential to avoid downstream clogging of the microfluidic device. During blood centrifugation, bacteria are enriched into the supernatant (see SI section “Cell and fluid movement during smart centrifugation” for a detailed description of bacterial cell trajectories during blood sedimentation). However, a large fraction of the bacteria are trapped in the plasma remaining in the blood cell sediment. Our strategy to avoid such bacterial loss is to layer the sample on top of a high-density medium with a volume sufficient to replace all plasma in the sediment. We adjusted the volumes and densities of both the sample and the density medium to achieve optimal bacterial isolation efficiency with minimal dilution. To tune the densities of the blood sample and density medium, we used blood culture medium (BCM) to support bacterial growth throughout the assay.

The densities of RBCs, WBCs, and platelets are in the ranges 1.086–1.122 g/ml, 1.057–1.092 g/ml and 1.072–1.077 g/ml^47^, respectively. As density medium, we chose a 2:1 volumetric mixture of Lymphoprep (STEMCELL Technologies, Canada) and BCM, which has a density of 1.051 g/ml. This mixing ratio increased the medium’s density, improving particle separation during sedimentation, while remaining low enough to allow even the least dense blood cells to settle into the medium, thereby ensuring effective separation of all blood cells from the plasma. The volume of the density medium was experimentally tuned to be small but sufficient to replace all plasma remaining between the sedimented cells. Diluting the blood sample with 25% BCM ensured that the sample’s density was below the density of the medium used for separation while only slightly reducing the bacterial concentration. Centrifuging time and force were experimentally tuned for optimal enrichment of *E. coli* in the supernatant while removing at least 99.8% of the RBCs. Details of our experimental deduction of these parameters are provided in SI.

**Fig. 2:**
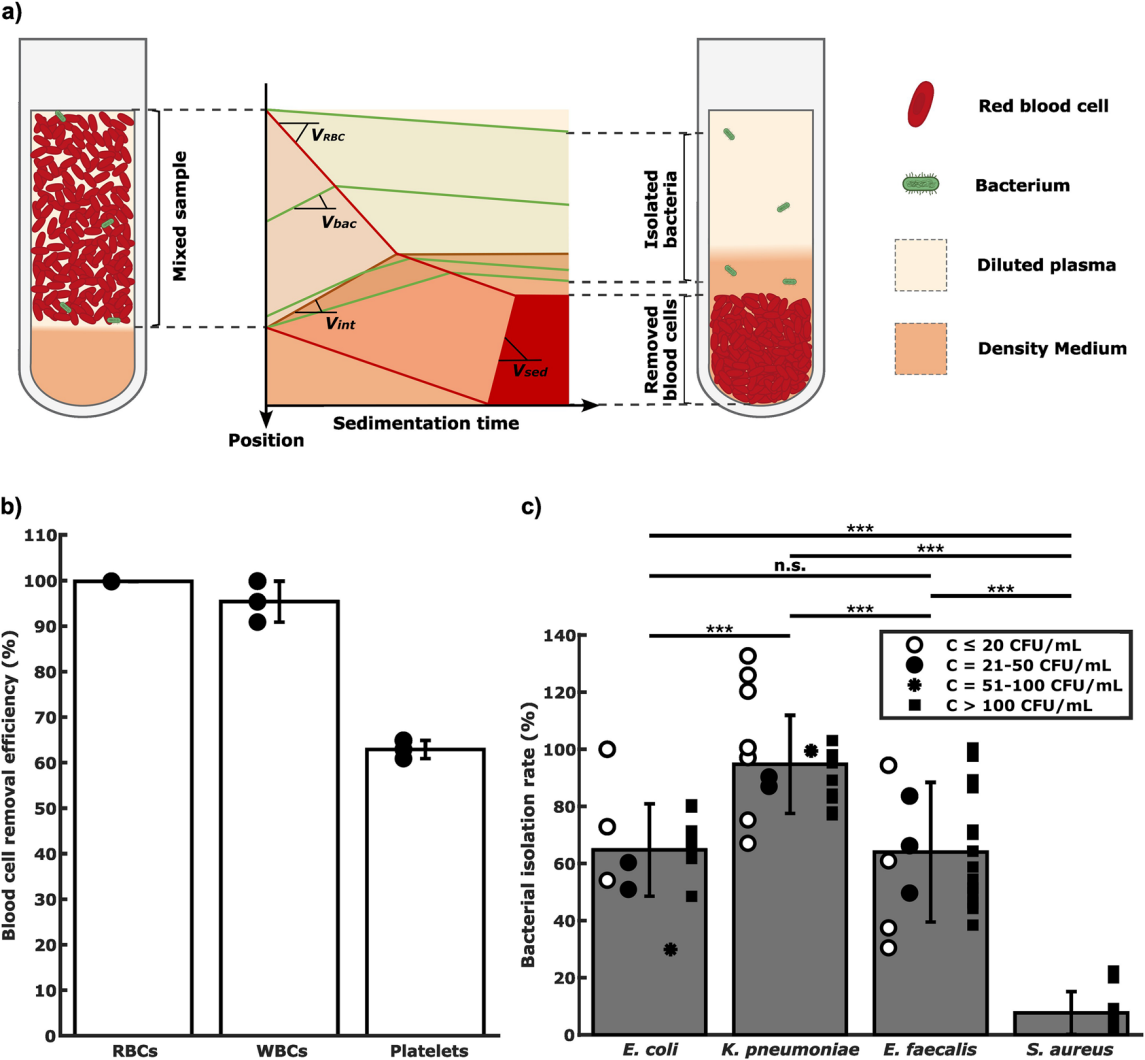
Bacterial isolation from blood by smart centrifugation. **a** *Illustration of liquid and cell movement*. The left and right tubes illustrate the positions of sample liquid, density medium, red blood cells and bacteria before and after smart centrifugation. The middle graph qualitatively illustrates the trajectories (solid lines) of bacteria (green) and red blood cells (red) during centrifugation, from a mixed state (left brackets) to a separated state (right brackets). The slopes of the lines are the particle sedimentation speeds, *v*_*RBC*_ and *v*_*bac*_, and liquid interface, *v*_*int*_, and sedimentation interface velocity, *v*_*sed*_, derived in SI. **b** *Blood cell removal efficiency*, meaning the fraction of blood cells removed from the supernatant after centrifugation relative to the initial number of blood cells in the sample (n=3). **c** *Bacterial isolation efficiency*, meaning the number of colony-forming units in the supernatant after centrifugation relative to the initial number of colony-forming units in the spiked sample. Bar heights are mean; error bars are sd; n.s. and *** indicate significance levels *p* > 0.05 and *p* ≤ 0.001, respectively; the bacterial concentration *C* refers to the CFU /ml in the blood sample. Each data point corresponds to an individual experiment. A total of *n* = 70 samples were tested. The figures were created and edited using the open-source software Inkscape (**a**) and MATLAB R2021 (**b**) and (**c**).

The optimized procedure involved layering 3 ml of BCM-diluted spiked blood on top of 1 ml density medium and centrifuging for 5 min at 600 × *g* in a hanging bucket centrifuge. The relative movements of the RBCs, bacteria, and liquid in such a system are described with a linear model in SI Section “Cell and fluid movement during smart centrifugation” and illustrated in Fig. 2a. After centrifugation, ~2.5 ml of clear supernatant containing most bacteria (SI Fig. 4) could be removed for further processing.

By counting the blood cells in the supernatant, we showed the we could remove 99.82 ± 0.04% of the RBCs, 95 ± 4% of the WBCs, and 63 ± 2% of the platelets (mean ± sd, *n* = 3; Fig. 2b). By plating the supernatant on agar plates and counting the resulting colonies, we demonstrated that, out of the spiked bacteria, we could recover 65 ± 16% of *E. coli*, 95 ± 17% of *K. pneumoniae*, 64 ± 24% of *E. faecalis*, or 8 ± 7% of *S. aureus* (mean ± sd, *n* = 10–26; Fig. 2c). Recovery exceeding 100% can be attributed to low colony counts, leading to considerable variations^56^.

### Selective blood cell lysis

In the second step of the assay, the remaining blood cells in the sample are removed by selective lysis using a mixture of sodium cholate hydrate and saponin^57^. Approximately 2.5 ml supernatant from the smart centrifugation step was mixed with 1 ml of the selective lysing solution and kept in a shaking incubator at 37 °C for 10 min, completely lysing remaining RBCs, WBCs, and platelets. Previous studies indicate a limited effect of the lysing solution on bacterial viability^57^.

### Volume reduction

The third assay step enriches the sample and removes the excess lysing buffer in a second centrifugation step. The sample-lysate mixture was layered on top of 0.3 ml high-density liquid, consisting of a 1:2 volumetric mixture of percoll and BCM, and centrifuged for 13 min at 1000 × *g* to sediment the bacterial cells on the liquid interface. The supernatant was removed, leaving ~0.5 ml liquid containing sedimented bacterial cells, as well as some blood lysate. Sedimenting the sample into a higher-density liquid resulted in a smoother liquid flow in the microfluidic chip during downstream processing (SI Fig. 5), while not significantly affecting the bacterial isolation efficiency (*p*-value >0.05) (SI Fig. 6).

### Bacterial microfluidic trapping

In the last step, we loaded the 0.5 ml bacterial sample on a microfluidic analysis chip at a pressure of 1 bar, keeping all outlets at atmospheric pressure. The design of the chip is the one introduced by Baltekin et al.^39^ for cross-flow filtration of bacteria into individual filter traps. We ran the resuspended sample through the microchip for 70 min to allow the bacteria to enter the traps. The chips contained in total 8000 microtraps of length 50 μm, height 1.25 μm and width 1.25 μ. A 300 nm restriction at the end of the trap prevents the bacteria from escaping into the back channel while still allowing liquid to flow over the cells. This design makes each microtrap function as a miniature culture chamber. The microtraps were monitored with fluorescence-microscopy to detect *E. coli*; alternatively phase-contrast microscopy to detect the clinical isolates of *K. pneumoniae*, *E. faecalis*, and *S. aureus*.

For *E. coli*-spiked samples, ~30% of the sample flowed through the traps with a typical filtrate flow rate of 1–2 μl/min ((SI Fig. 5b) and (SI Fig. 7a)). The trapping efficiency, meaning the number of positive traps, *N*, relative to the number of CFUs after smart centrifugation, was 29 ± 6% (mean ± sd, *n* = 5; SI Fig. 7b). The overall detection rates (%), meaning the number of cells observed on chip relative to the number of CFUs in the initial sample, ranged from 5 to 44%, depending on the bacterial species, as shown in SI Fig. 8.

### Microfluidic trapping efficiency

We evaluated the entire assay with blood spiked with *E. coli*, *K. pneumoniae*, and *E. faecalis*, respectively. All experiments resulted in successful trapping and subsequent detection of bacteria.

**Fig. 3:**
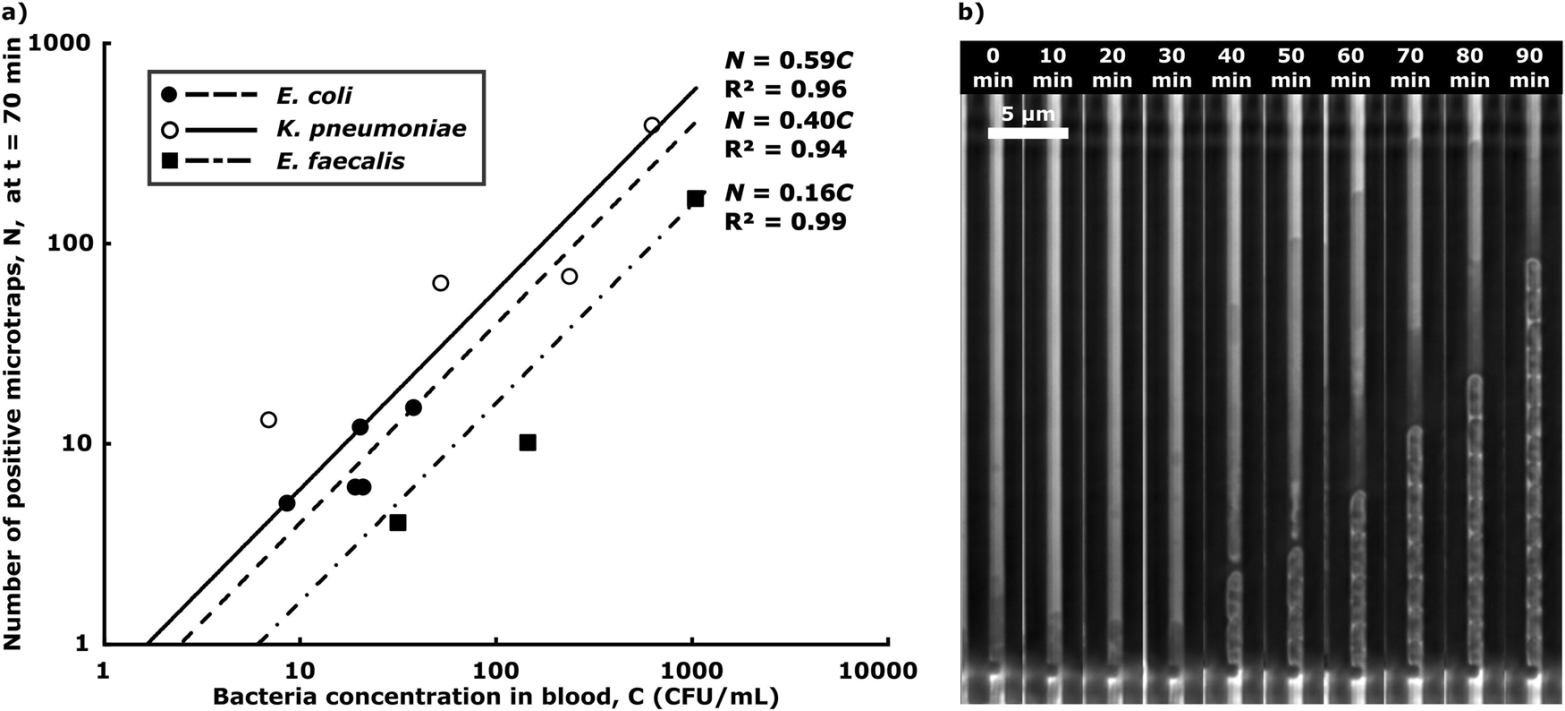
Bacterial microtrapping. **a** *Overall assay performance*, where dots represent the number of positive microtraps detected, *N*, for various bacterial concentrations in the 2.25 ml blood sample, *C*, and lines depict the least square fitting linear calibration curves *N* = *η* ⋅ *C*. Each point corresponds to an individual experiment. A total of *n* = 12 samples were tested. **b** *Timelapse images of bacterial capture and growth in a single microtrap*, showing the capture of one bacterium of *K. pneumoniae* 40 min after sample addition, followed by bacterial cell division. The figure was created with Microsoft Excel and edited and assembled using the open-source software Inkscape.

The lowest bacterial concentrations, *C*, tested and detected were 9, 7 and 32 CFU/ml, respectively (Fig. 3a). Fitting a least mean square linear curve, *N* = *η* ⋅ *C*, to the data allowed us to estimate the overall assay sensitivity, *η*. We used the data collected from the first manual labeling of the image data set, to avoid data bias. We could infer that the detection limits, which are the bacterial concentrations for which we expect *N* = 1 positive trap, *C*_*N*=1_ = 1/*η*, were in the range 1–10 CFU/ml. After trapping, the bacterial cells divide inside the microchannels, while the sample is still being loaded (Fig. 3b).

Due to poor isolation efficiency of the smart centrifugation step, we could not detect *S. aureus* (*n* = 2).

### Deep-learning based detection of bacteria

We trained deep learning models to automatically detect the presence of bacteria in 8-frame (70 min) 1300 × 40 pixel (height, width) time-lapse image series of microfluidic traps, posing the detection as a video classification problem. The dataset was collected through phase-contrast microscopy, capturing bacterial growth in the microfluidic device and then using a pre-processing pipeline to find and crop out individual traps. Each image of every time-lapse was manually labeled, tagging the presence of bacteria in each frame. The training set totaled 57,608 time-lapses (24,220 positives and 33,388 empty at 70 min; SI Table 4), and the test set 66,869 time-lapses (721 positives and 66,148 empty at 70 min; SI Table 5), of data from eight different experiments. A trap has a positive label if it contains or previously contained any frames with bacteria until the current evaluation time point. The number of positive instances changes over time as cells get trapped in previously empty traps, shown in SI Table 6. Additionally, we performed negative control experiments (*n* = 3), using non-spiked sterile blood, each containing a total of 8000 traps, to further evaluate the specificity of the detection.

**Fig. 4:**
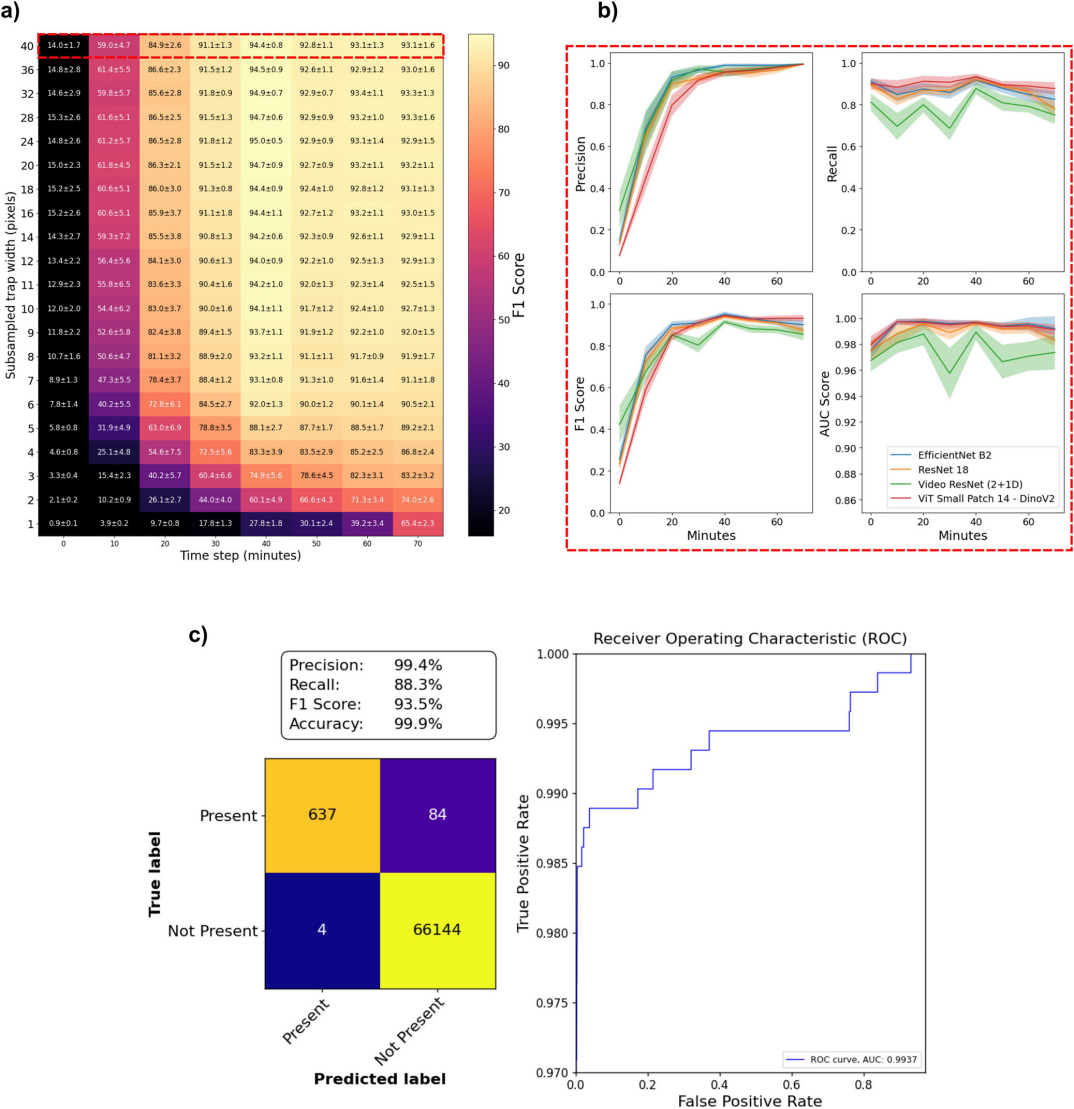
Deep learning model performance. **a** Evaluating networks using downsampled data, testing on an increasing number of frames (each corresponding to 10 min). The heatmap shows the F1-Score of DinoV2. **b** Precision, recall, AUC (Area under the curve), and F1-Score of all models over time at full resolution, corresponding to the red dashed rectangle in part **a** Lines show mean, and shaded areas show standard deviations among the 30 retrainings. **c** Confusion matrix, performance metrics, and ROC (Receiver Operating Characteristics) curve of the DinoV2 model with the median AUC score among the 30 retrainings, at full resolution, evaluated at the final time (70 min). The figure was created using the open-source software Inkscape and the Python plotting library Matplotlib.

First, time-lapse frames were concatenated horizontally to a frame montage, allowing us to use image classification networks for the video detection task. We trained and compared three similarly sized models: ResNet 18^58^ from 2016 (11.2M parameters, 8.4 GFLOPs), EfficientNet B2^59^ from 2021 (7.7M parameters 3.2 GFLOPs), and the more modern foundation model DinoV2 Small Patch 14^60^ from 2023 (21.8M parameters 24.8 GFLOPs). DinoV2 is a standard vision transformer, ViT^61^, trained using self-supervision on a large dataset with the DinoV2 methodology; for simplicity, we hereafter refer to it as DinoV2. Then, we employed the Video ResNet R(2+1)D^62^ from 2018 (31.3M parameters 47.5 GFLOPs), processing the unaltered time-lapse video directly. The evaluation was conducted by progressively increasing the number of frames in the test time-lapse movies, measuring the performance over time (0–70 min) (SI Fig. 11a). Also, we trained and tested the models using spatially downsampled images to assess the complexity of the task and evaluate how much performance remains as image detail is progressively lost (SI Fig. 11b). Each network and downsampling step was retrained 30 times with a specific random seed for reproducibility and better statistics. The evaluation metrics used were precision, defined as: $\frac{\,{{\rm{True}}\,{\rm{Positives}}}}{{{\rm{True}}\,{\rm{Positives}}}+{{\rm{False}}\,{\rm{Positives}}}\,}$, recall (sensitivity), defined as: $\frac{\,{{\rm{True}}\,{\rm{Positives}}}}{{{\rm{True}}\,{\rm{Positives}}}+{{\rm{False}}\,{\rm{Negatives}}}\,}$, and the F1-score, which is defined as the harmonic mean of precision and recall: ${F}_{1}=2\cdot \frac{\,\text{precision}\cdot \text{recall}}{\text{precision}+\text{recall}\,}$. Given the large test set imbalance, we chose the F1-Score as an evaluation metric because this method takes into account both the precision and recall. Additionally, ROC (Receiver Operating Characteristic) curves and AUC (Area Under the Curve) scores were calculated. At 70 min, the mean and standard deviation F1 scores were 85.5 ± 2.8% for Video ResNet R(2+1)D, 87.3 ± 2.2% for ResNet 18, 90.1 ± 2.3% for EfficientNet B2 and 93.1 ± 1.6% for DinoV2, respectively (full resolution). Figure 4 shows the confusion matrix, downsampling, and time evaluation heatmap for DinoV2 (the best-performing model), along with the time evaluation classification metrics for all models at full resolution. For the negative control tests with sterile blood, the results showed that all video detection models reported zero bacteria detections at time 70 min for all tests (*n* = 3) (SI Fig. 17, see SI section “Negative control tests”). The inference latency for each model is outlined in SI section “Inference times and computational complexity”. Some cases of obviously incorrect manual labeling of the test data were corrected. For details, see SI section “Data cleaning and label adjustments”.

We also analyzed the image series by performing inference on each frame separately, disregarding any temporal or sequential dependencies that might exist between consecutive frames. This approach, which is outlined in SI section “Posing the problem as single-frame image classification", is feasible and performs well, as shown in SI Fig. 15. However, in the negative control experiments, false positives occurred throughout the whole time-series, as shown in SI Fig. 18.

## Discussion

We developed and tested a five-step assay to isolate and detect bacteria in blood samples without pre-culture. We show that we can successfully use the assay to trap and detect three sepsis-causing bacterial species, *K. pneumoniae*, *E. faecalis*, and *E. coli*, which cover ~20% of the bacterial species causing BSIs in the European Union^63^ and 35% of those in Japan^64^. The total assay time is below 2 h, which includes 5 min for the first centrifugation, 10 min for lysing, 13 min for the second centrifugation and 70 min for the microfluidic trapping. Automatic image analysis, based on deep-learning, occurs in less than 30 s for the whole data set. The current hands-on time is ~10 min. The estimated detection limit in the range 1–10 CFU/ml is clinically relevant. Compared to previous work, our approach does not require blood culture and allows the detection of bacteria phenotypically at clinically relevant concentrations. For *S. aureus*, the poor isolation efficiency lead to detection failure.

Smart centrifugation is a simple, inexpensive, high-throughput, and easily parallelizable method to rapidly isolate bacteria from whole blood with a high bacterial isolation efficiency and blood cell rejection. The method’s compatibility with standard lab equipment supports translation to the clinical setting. The bacteria are isolated in a small volume of liquid, similar to the original plasma volume, which facilitates downstream processing.

Two distinct mechanisms contribute to the enrichment of bacteria in the supernatant during centrifugation. First, the Stokes terminal velocity of bacteria is a factor *α* ≃ 1/30 times that of blood cells, allowing efficient terminal velocity-based differentiation.^47^ Second, the high hematocrit results in downward sedimenting RBCs causing a backflow of the plasma, thereby dragging bacteria upward and further increasing differentiation between the two. Smart centrifugation enhances the separation by extending the sedimentation path of particles through the density medium. For comparison, we experimentally optimised isolation of *E. coli* from spiked whole blood in conventional centrifugation, i.e., without sample dilution or density medium, and achieved 34% ± 7% isolation efficiency in the supernatant (SI Fig. 2). Smart centrifugation thus provides a 65%/34% = 1.9 times improved isolation efficiency for *E. coli* for a comparable blood cell rejection efficiency (SI Table 3).

Even though our method was optimised for *E. coli*, isolation efficiency of *K. pneumoniae* was significantly higher. Isolation efficiency of *E. coli* and *E. faecalis* were not significantly different, but isolation efficiency of *S. aureus*, however, was much lower. We hypothesize that several factors can cause the recovery differences between the species, as well as the large variation in recovery between different measurements with the same strain. These factors include differences in Stokes radius between the bacterial species^47^, differences in interaction between the blood cells and the bacteria, and differences in viscosity. We ascribe the variability in the isolation efficiency data to biological variability in patients and bacteria, and to technical factors. The blood samples used in the study were obtained from different donors, introducing biological variability, particularly in hematocrit levels, which can range from 40 to 54% in males and 36 to 48% in females^65^. Since smart centrifugation relies on density-driven sedimentation, such hematocrit differences can directly affect the separation efficiency and bacterial recovery. Another important source of variability arises from the use of low bacterial concentrations, especially close to the detection limit. At these low CFU levels, differences in individual bacteria behavior are amplified. Furthermore, plate counting becomes more susceptible to statistical fluctuations, further increasing measurement uncertainty and contributing to variability in recovery^56^. Additional sources of variability may include variations in the immune response of the patient blood to the bacteria (reducing the bacterial count), or variations in bacterial growth phase (compared to *K. pneumoniae*, *E. coli* were spiked in stationary phase) or growth rate (*E. faecalis* have a slower growth rate than *K. pneumoniae*). We found no significant differences in bacterial isolation efficiencies between samples spiked with the same strain at different concentrations (SI Fig. 1), with the exception of *E. coli*, where we found a significantly lower isolation efficiency for concentrations from 21 to 100 CFU/ml compared to >100 CFU/ml (*p* ≤ 0.05). However, the statistical power of this finding is low considering the very low number of measurements.

The behavior of *S. aureus* in blood has been extensively studied previously^66^. *S. aureus* secretes two coagulases, Coagulase (Coa) and von Willebrand factor binding protein (vWbp), which hijack the host coagulation cascade and trigger the formation of fibrin clots and/or blood clots, in which the bacteria hide from the effects of the immune system and thereby increase their survival^67–69^. We speculate that the poor isolation efficiency of *S. aureus* in our approach is caused by this phenomenon, in which clots containing bacteria rapidly sediment together with the blood cells. Indeed, experiments in which *S. aureus* were spiked in a sample liquid with the same density as diluted blood but without blood cells resulted in 54 ± 6% (*n* = 6) isolation efficiency by smart centrifugation, compared to 8 ± 7 % (*n* = 10) isolation efficiency from spiked blood (SI Fig. 3). To approach this challenge, some literature suggests adding the anti-coagulant and direct thrombin inhibitor Argatroban Monohydrate to the blood^48^. Although this drug has been clinically proven to improve outcomes in patients treated with it and that had previous positive *S. aureus* blood cultures^70^, we argue that its use post-sampling would not be successful, as clot formation occurs inside the patient, pre-sampling, and the drug cannot reverse clotting. Poor isolation of *S. aureus* remains an ongoing challenge and we suggest future research to focus on alternative fibrinolytic agents. Based on epidemiological data, we estimate that this limitation could lead to false negatives in ~10% of sepsis cases, where *S. aureus* or *MRSA* are the causative agents^63,64^. Other common sepsis-causing pathogens, including *E. coli*, *K. pneumoniae*, *E. faecalis*, *S. pneumoniae*, *S. pyogenes*, *P. aeruginosa*, Candida species, and other Enterococcus species, do not exhibit the same coagulase activity and are therefore not expected to pose similar isolation challenges.

Based on our reading of the literature, *Smart centrifugation* has superior recovery and throughput for *E. coli*, data for which are presented for most tests (SI Table 3).

The concentrated bacterial sample was loaded onto the microfluidic chip at 1000 mbar. At higher pressures, we observed bacterial loss through the 300 nm constrictions of the microtraps. Sedimenting into a higher-density liquid during the prior volume reduction step reduced the compression of bacterial cells and lysate and allowed more homogeneous resuspension, leading to lower on-chip clogging.

The trapping of bacterial cells and blood cell debris in the traps increases the fluidic resistance of the filter and decreases the through-filter flow over time. The chips enabled processing 0.5 ml sample within 70 to 90 min, during which 30% of the sample flows through the microtraps (filtrate flow) and the rest flows past the filter directly to the waste outlet (retentate flow). The low filtrate flow rate is thus a major contributor to bacterial loss between isolation and detection.

One limitation of the current assay is the risk of false negatives at very low bacterial concentrations due to an overall detection yield of 5–44%. To mitigate this, two strategies could be employed: (1) increasing the processed blood volume from 2.25 mL to 10 mL to enhance bacterial capture probability, and (2) introducing a brief pre-incubation step (2–3 h) to allow bacterial enrichment before processing. As antibiotics are typically administered in 6-h intervals, such enrichment would still support timely clinical decision-making. A potential limitation of this would be the detection of organisms that do not exhibit growth, which would have to be investigated in the future.

The deep learning experiments demonstrate the feasibility of using deep neural networks to accurately detect the presence of bacteria in phase-contrast time-lapses. Both transformer-based models and convolutional neural networks achieve similar performance. The task can also be framed as single-frame image classification with good results. However, in a real-world scenario, it would be more advantageous to use the entire time-lapse sequence to detect bacterial growth, especially if using low-resolution microscopy. This is also demonstrated by our negative control experiments, where single-frame models produce more false positives in later time steps (see SI Fig. 17) due to their inability to detect actual growth.

This proof of concept demonstrates that it is possible to train a classifier from a few experiments with good performance. Future work could stratify classification metrics by species, although it remains unclear whether significant differences in classification difficulty exist between species. Inference times for a single time-lapse (corresponding to one micro-trap) ranged from 1 to 3 ms on our system, indicating that latency should not pose a significant issue, even on automated microscopy systems with less powerful hardware.

In summary, our approach offers a rapid, culture-free approach for detecting bacteria directly from blood at clinically relevant concentrations within 2 h. The overall hands-on time is ~10 min and could be reduced through future robotic automation for further clinical implementation. The experimental workflow, encompassing smart centrifugation, selective lysis, and microfluidic trapping, is supported by automatic image analysis that yields results regarding bacterial presence in under a second. We suggest future work to focus on clinical validation of the assay, as well as incorporation of species identification^71,72^, and phenotypic-AST at the single-cell level^39,73^, for which the microfluidic platform is intrinsically suited. Finally, for seamless clinical incorporation, robotic automation will be required to further reduce operator time, making this assay a powerful candidate for routine, high-throughput sepsis diagnostics.

## Methods

### Microfluidic platform fabrication

The microfluidic chip design and fabrication were previously reported^39,74^. A silicon wafer mold was fabricated by the company ConScience AB, Sweden. The mold was fabricated on a 6-inch (100) silicon wafer. A thermal oxide layer was first grown to a thickness matching the height of the nanochannels. An etch mask for the nanochannels was then patterned using electron beam lithography, and the nanochannels were etched into the oxide using reactive ion etching. The microchannel structures were subsequently patterned in photoresist by laser lithography, and the photoresist defining the microchannels was reflowed. The wafer was once silanized for 30 min prior to polydimethylsiloxane (PDMS; Sylgard 184, DOW, USA) replication. Subsequently, a 10:1 w/w PDMS:curing agent mixture was poured on the wafer and baked at 80 °C overnight. Holes on the PDMS ports 2.0, 2.1, 2.2, 5.1, and 5.2 (see chip design in refs. ^39,74^) were made using a 0.5 mm puncher. Then, the PDMS stamps were cleaned in IPA before covalent bonding to a glass coverslip (No. 1.5, Menzel-Gläser, Germany) after plasma treatment, followed by heat curing for 1 h at 80 °C. Approximately 10% of the devices fabricated were discarded due to the presence of dust in the channels of the PDMS stamp, derived from the microfabrication process.

### Microfluidic flow control

The microfluidic device was mounted on the microscope and connected to the sample reservoirs with flexible plastic tubing (TYGON, Saint-Gobain, North America). OB1 CONTROLLER (Elveflow, France) flow control units were used to pressurize reservoirs.

The reservoirs connected to ports 2.1, 2.2, 5.1 and 5.2 were first pressurized at 500 mbar for chip priming with water infused with 0.085 g/l of Pluronic F108 (Sigma-Aldrich, USA) and was decreased to 0 mbar. Then the reservoir connected to inlet port 2.0 was pressurized at 500 mbar for priming and at 1000 mbar for sample loading.

### Blood sample preparation

Blood in EDTA tubes from healthy donors was purchased from the blood bank (Blodcentralen, Stockholm, Sweden) or Uppsala University Hospital (Akademiska Sjukhuset, Uppsala, Sweden). The blood samples were stored at 4 °C and used for experiments no later than two days after collection. Samples presenting a milky supernatant (<10%), i.e., indicating abnormally high concentrations of triglycerides in the blood, were discarded. Before processing the samples, they were put at room temperature, and blood samples were spiked with respective bacteria concentrations of approximately 10^3^, 10^2^, and 10 CFU/ml. The concentration of the spiking solution was quantified by plate counting (*n* = 3). The spiking volume was always less than 5% of the total blood solution volume.

We used anonymized blood obtained from healthy donors through a certified blood bank, solely for technical development purposes, and therefore, no specific ethical approval was required under Swedish regulations.

### Bacterial strains

We used three clinical isolates that were randomly collected from a clinical microbiology laboratory in Sweden, covering both gram-negative and gram-positive species. As gram-positive representatives, *S. aureus* (DA70300) and *E. feacalis* (DA70208) were used, and as gram-negative, *K. pneumoniae* (DA72206). We also used *E. coli* (EL3041) cells harboring a plasmid expressing mVenusNB fluorescence proteins. For the machine learning detection experiments, a non-fluorescent strain of *E. coli* (EL330) was used.

Bacteria were stored for long term at –80 °C in standard glycerol solution and were incubated overnight at 37 °C in BD BACTEC Plus Aerobic medium (BD, USA), referred to as Blood Culture Medium (BCM), prior to use, and later diluted to approximately 10^4^, 10^3^, and 10^2^ CFU/ml and used to spike blood with different concentrations and quantified for isolation. For detection experiments with *E. coli* cells harboring a plasmid expressing mVenusNB fluorescence proteins, the above-mentioned protocol was followed. For detection experiments with clinical isolates and non-fluorescent *E. coli*, 2 μL of bacteria from an overnight culture were spiked in 2 ml of BD BACTEC Plus Aerobic Medium, and cultured for 2–4 h depending on the species. They were then diluted to approximately 10^4^, 10^3^, and 10^2^ CFU/ml and used to spike blood with different concentrations.

### Smart centrifugation

Lymphophrep (STEMCELL Technologies, Canada), a density medium with 1.077 g/ml density, was mixed with BD BACTEC Plus Aerobic medium (BD, USA) in a volumetric ratio of 2:1, and 1 ml of the solution, with 1.051 g/ml density, was poured into a 15 ml Falcon centrifuge tube. 3 ml of spiked blood mixed with BD BACTEC Plus Aerobic medium (BD, USA) (3:1 Blood:BD BACTEC Plus Aerobic medium) was gently placed over the density media. The tube was centrifuged at 600 × *g* for 5 min in a hanging bucket centrifuge. The supernatant was removed and mixed with the lysing solution.

### Conventional centrifugation for bacteria isolation

To establish a control measurement, we centrifuged 4 ml of whole blood without any dilution and density media. After being spiked, the sample underwent centrifugation at 500 × *g* for 4 min, resulting in the separation of clean plasma at the top. This process effectively settled most of the blood cells.

### Selective lysing solution

The selective lysing solution was prepared by mixing sodium chocolate hydrate (Sigma-Aldrich, USA) and saponin (Sigma-Aldrich, USA). Concentrations of 2% (W/V) sodium cholate hydrate and 1% (W/V) saponin were prepared by dissolving the chemicals in BD BACTEC Standard Aerobic medium.

### Volume reduction

Percoll (Sigma-Aldrich, USA), a density medium having a density of 1.125–1.135 g/ml was mixed with BD BACTEC Plus Aerobic medium (BD, USA) in a volumetric ratio of 1:2, and 0.3 ml of the solution, with ~1.04 g/ml density, was poured into a 15 ml Falcon centrifuge tube. The supernatant infused with the lysing solution was gently placed over the density medium. The tube was centrifuged at 1000 × *g* for 13 min in a fixed rotor centrifuge. All excess liquid above 0.5 ml was removed, without removing the pellet.

### Optical setup

Images of all the microtraps contained in the microfluidic device were acquired using a Nikon Ti, inverted microscope. For imaging experiments containing clinical strains, a CFI plan Apo lambda 100x (1.45 NA, oil) objective was used. Phase contrast images were acquired by using DMK 38UX304 (the imaging source) camera, with an exposure time of 20 ms with an interval of 10 min, for a maximum of 90 min. Cells expressing mVenus fluorescence proteins were captured using a filter cube consisting of a Di02-R488 (Semrock, USA) dichroic mirror, a FF02-482/18 (Semrock, USA) excitation filter, and a FF01-524/24 (Semrock, USA) emission filter and CFI Plan Apo lambda DM Ph2 20x objective. Each fluorescence image was acquired with an exposure time of 500 ms with an interval of 5 min, for a maximum of 90 min. A constant temperature of 37 °C was maintained during the measurements using a temperature-controllable unit (Okolab, Italy). The imaging setup was operated by micromanager 1.4 version software.

### Bacteria quantitation

All bacterial sample quantitation was performed by plate counting after plating a sample aliquot on agar plates and overnight culture at 37 °C in the incubator. The agar plates were prepared by dissolving LB broth with agar (Miller) (Sigma-Aldrich, USA) in deionized water at 40 g/L concentration followed by autoclaving and placing in Petri dishes.

For on-chip bacteria quantitation of the clinical isolates, we employed ImageJ software image analysis of phase-contrast microscopy images. Each microtrap was analyzed manually and determined positive if filled with at least one bacterium. The same analysis pipeline was performed for fluorescence microscopy images of the *E. coli* cells expressing mVenus fluorescence proteins.

### Blood cell quantitation

The concentrations of the blood cells in whole blood before dilution and in the supernatant after smart centrifugation were measured using a hematology analyzer (Swelab Alfa Plus, Boule Diagnostics, Sweden).

### Flow characterization

The ports 2.1 and 2.2 were coupled together, and ports 5.1 and 5.2 were coupled together, using a PEEK union T-connector (Thermo Fisher Scientific, USA). Flow measurements were taken from both couplers using a SLI Liquid Flow Sensor (80 μL/min) (Sensirion, Switzerland).

Two independent flow measurements were acquired with a sampling time of 1 s, for a maximum time of 90 min, using the Sensor Viewer Software (Sensirion, Switzerland). The data was analysed using MATLAB R2021.

### Image cropping and stabilizing

An image processing pipeline was further improved from ref. ^71^ to rotate, find, and crop out 1300 × 40 pixel trap images in the microfluidic chip, and then stabilizing the corresponding time-lapse described in SI section “Preprocessing and cropping”. During deep learning model training and inference, the frames were cropped vertically at 336 pixels from the physical stop at the top of each trap and padded to a width of 42 pixels. The reason for padding the traps to 42 pixels width is that we hypnotize DinoV2 would perform better, as it uses patches of 14 × 14 pixels for the token embeddings, meaning it would fit three tokens on the width of a trap. The rationale for the 336-pixel cropping of the height was to fit the time-lapses in the GPU memory and concerns of overfitting. Additionally, the difficult instances had one or a few cells situated at the top adjacent to the physical stop, thus, processing the whole trap was unnecessary.

### Time-lapse images and downsampling

When employing image classification models (ResNet 18, DinoV2, and EfficientNet B2), the frames of each trap were concatenated horizontally to a 336 × 336 pixel 2D image showing the time-lapse. Zero-padding was applied on the right side when performing the time evaluation, shown in SI Fig. 11. Padding was also applied during training if the time-lapses from the experiment contained less than eight frames, seen in SI Table 4. For the Video ResNet R(2+1)D, we instead relied on the global averaging pooling^75^ to test and train on time-lapses with different numbers of frames. A time-lapse sample had a positive label if it contained any positive frames to the evaluated time-point (also if cells had been previously trapped and subsequently escaped). Lancoz^76^ downsampling was used to test under degraded image conditions and test how much high-frequency information can be removed without degrading performance. Before feeding the downsampled images into the model, they were upsampled to their original size using bicubic interpolation. This step was necessary because DinoV2 is a transformer with a fixed patch size, thus, it could not process images with smaller widths than 14 pixels. The single-channel grayscale phase-contrast data was transformed to RGB using the Vidiris color map^77^ for visibility and to aid human labeling. The transformed color images were also used when feeding into the networks (we kept the 3-channel input).

### Network training

All models were trained for 20 epochs with batch size 32 and learning rate 10^−3^ except DinoV2 (batch 128, learning rate 10^−5^, trained for 15 epochs) and Video ResNet R(2+1)D (used learning rate 10^−4^). All networks used pretrained weights, employed the standard ADAM optimizer^78^, Cross Entropy loss, Cosine learning rate scheduler^79^, and 15% label smoothing^80^. The Pytorch deep learning framework was used with Pytorch Image Models (Timm)^81^. During training, images were sampled with replacement for each mini-batch from the two classes, with sampling probabilities inversely proportional to the class overrepresentation in the training set (using a Random Weighted Sampler in Pytorch). The augmentations ShiftScaleRotate, HorizontalFlip, VerticalFlip, RandomBrightnessContrast, and Blur were employed during training from the Albumetations library^82^. Additionally, 1-7 frames (sampled uniformly) were randomly erased during training (random frame erase augmentation). All models were trained on the NvidiaA100 GPU.

For further information, refer to the released software package containing the dataset and software to reproduce the deep learning experiments.

### Statistical analysis

Data analyses were performed with R and Microsoft Excel. The data samples were tested for normality using the Shapiro-Wilk test; variance between normal populations was studied using the F-test; and statistically significant differences between groups were studied using a *t*-test, for samples following a normal distribution, or a Mann–Whitney test for samples not following a normal distribution. A two-sided *p*-value of ≤0.05 was considered statistically significant.

## Supplementary information

Supplementary information

## Data Availability

The raw data (microscopy image data, flow measurements, plate count data, analysis summary files, codes, etc.) used to generate the information compiled in this manuscript can be found in the following open repository: https://figshare.com/s/affea349261af5a2f637.

## Code availability

All codes and replication packages for the ML models can be found in the following open repository: https://figshare.com/s/affea349261af5a2f637.
