# Supplementary material for: Culture-free detection of bacteria from blood for rapid sepsis diagnosis
Source: NPJ Digit Med. 2025 Aug 25;8:544. doi: 10.1038/s41746-025-01948-w (PMC12375708; doi:10.1038/s41746-025-01948-w)
Supplement: Supplementary file 1 — Supplementary information [file 41746_2025_1948_MOESM1_ESM.pdf]

Supplementary information

Cell and fluid movement during smart centrifugation

The movement of blood cells during centrifugation has been studied extensively [1]. To understand the displacement of bacteria during centrifugation, we here consider a linear model for the simultaneous sedimentation of two types of particles, bacteria and RBCs, through two types of density-layered liquids, diluted plasma and density medium, in which we neglect particle-particle interactions. This manuscript uses the indices *bac*, *RBC*, *pl*, *DM*, *int*, and *sed*, to indicate bacteria, RBCs, plasma, density medium, liquid:liquid interface and sediment, respectively.

The theoretical stokes terminal velocity, v_{st}, of particles in blood can be deduced from Stoke's law, which describes the unhindered sedimentation velocity of a single particle in a standing fluid. The stokes velocity depends linearly on the density difference between the particle and the medium, $\Delta\rho$, quadratically on the particle size, and inversely linear on the viscosity, μ. The stokes velocities of a given particle in the two liquids thus relate as

$$\frac{v_{st,DM}}{v_{st,pl}} = \frac{\mu_{pl}.\Delta\rho_{DM}}{\mu_{DM}.\Delta\rho_{pl}}. \tag{1}$$

Because the densities of bacteria and RBCs are in the same range, the ratio of their stokes velocities can be considered a constant, α, independent from the liquid medium:

$$\alpha = \frac{v_{st,bac}}{v_{st,RBC}}. \tag{2}$$

α has been estimated to be typically 1/30 [2].

In regions with a large local volume fraction of RBCs, h, the RBC sedimentation causes a significant upward backflow of liquid medium. Considering RBCs incompressible, the local backward liquid velocity, v_l, can be deduced from mass conservation, i.e., the downward flux of RBCs must equal the backward flux of liquid:

$$h.v_{RBC} + (1-h).v_l = 0 \iff v_l = -\frac{h}{1-h}.v_{RBC}. \tag{3}$$

(Throughout this manuscript, we use a negative sign for velocities to indicate an upward movement direction opposite to that of downward sedimenting RBCs.) Where the sedimenting RBCs cross the liquid interface between the sample and density medium, mass conservation demands that the local liquid velocities across the interface, and the velocity with which the interface itself moves backward, are all equal. This equality implies a relation between the difference in local RBC fractions, h_{pl} and h_{DM}, across the liquid interface:

$$v_l = v_{int} = v_{pl} = v_{DM} = -\frac{h_{pl}}{1-h_{pl}}.v_{RBC,pl} = -\frac{h_{DM}}{1-h_{DM}}.v_{RBC,DM}. \tag{4}$$

The effective sedimentation velocity of the particles equals the sum of the local liquid velocity and their theoretical Stokes velocity. For RBCs, the resulting effective sedimentation velocity can be expressed as

$$v_{RBC} = v_l + v_{st,RBC} = -\frac{h}{1-h}.v_{RBC} + v_{st,RBC} \Longleftrightarrow v_{RBC} = (1-h).v_{st,RBC}, \tag{5}$$

i.e., $v_{RBC,pl} = (1-h_{pl}).v_{st,pl,RBC}$ and $v_{RBC,DM} = (1-h_{DM}).v_{st,DM,RBC}$.

The values for $v_{st,RBC}$ can be derived relative to those of blood using equation 1. The value of h_{pl} can be estimated from the hematocrit value while accounting for the 25 % blood sample dilution with BCM. The value of h_{DM} can then be derived from h_{pl} using equation 4.

During sedimentation, bacteria can assume any of four velocities, depending on their location in either the plasma or density medium, and on the local RBC fraction being zero or not.

$$v_{bac} = v_l + v_{st,bac} = -\frac{h}{1-h}.v_{RBC} + \alpha.v_{st,RBC} = -(h-\alpha).v_{st,RBC}. \tag{6}$$

In the supernatant and in density medium depleted from RBCs, $h = 0$ and $v_{pl} = 0$ and bacteria reach their stokes terminal velocity $v_{bac} = v_{st,pl,bac}$.

Importantly, and perhaps counterintuitively, bacteria move upwards where bacterial fractions $h > \alpha$.

The speed at which the sedimentation layer grows depends on the liquid fraction, β, remaining between the RBCs in the sediment:

$$v_{sed} = v_l.(1+\beta). \tag{7}$$

The values of v_{int}, v_{bac}, v_{RBC} and v_{sed} are visualised as the slopes of the position-versus-time trajectories indicated in Figure 2a)

Statistical Analysis of Smart Centrifugation of Samples with Different Bacterial Concentration

We have studied the variation in isolation efficiency of bacteria by smart centrifugation between the different concentration ranges < 20, 20-100 and >100 CFU/ml for three bacterial species (SI Figure 1). For *K. Pneumoniae* and *E. Faecalis* we found no significant difference. For *E. coli* we found a significant difference only in between the concentration ranges < 20 CFU/ml and 20-100 CFU/ml.

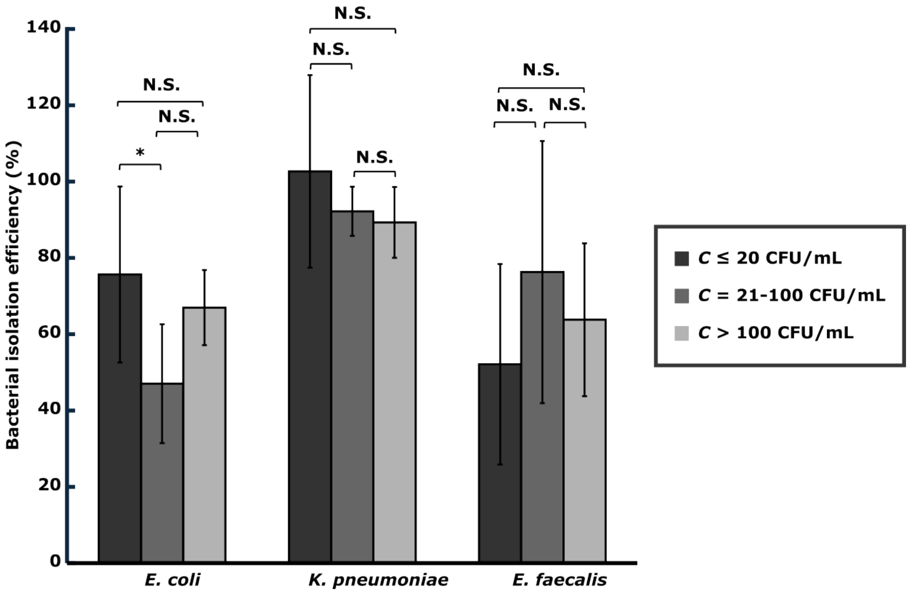

SI Figure 1: **Isolation efficiency of bacteria from blood by smart centrifugation in different concentration ranges.** n.s. and * indicate significance levels $p > 0.05$ and $p \leq 0.05$, respectively. n=70 blood samples were tested. The figure was created with Microsoft Excel and edited using the open-source software Inkscape.

Isolation of bacteria by conventional centrifugation of undiluted blood without density medium

The bacterial isolation efficiency for (*E. coli*) has also been studied for conventional centrifugation, i.e., centrifugation without sample dilution or density medium, of whole blood. 4 ml of whole blood was centrifuged at 500g for 4 min. 4 min was sufficient for most of the blood cells to settle. We recovered 34 ± 7 % (mean ± sd; n=3) of the bacteria in the supernatant (SI Figure 2), removing 99.957 ± 0.007% of the RBCs, 98.9 ± 0.6 % of the WBCs, and 65 ± 2 % of the platelets (mean ± sd; n=3) (SI Table 1).

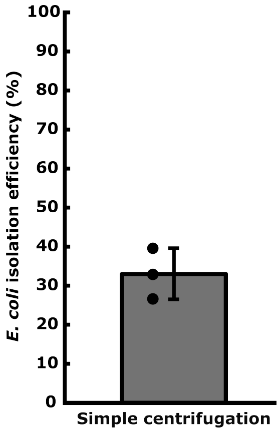

SI Figure 2: **Bacterial isolation efficiency in conventional centrifugation of blood (n=3).** The figure was created with Microsoft Excel and edited using the open-source software Inkscape.

SI Table 1: Data points for % rejection of blood cells in conventional centrifugation (n=3).

Cell type	% rejection (E1)	% rejection (E2)	% rejection (E3)	% Average rejection	SD
RBC	99.94	99.96	99.96	99.953	0.012
WBC	99.19	98.13	99.26	98.86	0.63
Platelets	62.76	65.86	67.22	65.3	2.3

Blood cell rejection in smart centrifugation

SI Table 2: Data points for % rejection of blood cells in smart centrifugation (n=3).

Cell type	% rejection (E1)	% rejection (E2)	% rejection (E3)	% Average rejection	SD
RBC	99.87	99.8	99.79	99.820	0.044
WBC	90.9	95.27	99.86	95.3	4.5
Platelets	63.27	64.68	60.71	62.9	2.0

Low isolation efficiency of *S. aureus*

We investigated whether the poor isolation efficiency of *S. aureus* is caused by their interaction with the blood cells or plasma protein, as reported by many, or whether the poor performance is intrinsic to smart centrifugation. We replaced the spiked blood in smart centrifugation with a control liquid having the same density and volume as the diluted sample, and performed smart centrifugation by placing it over 1 ml density media. After 5 min of centrifugation, the top 2.5 ml of liquid was removed and plated on an agar plate for bacteria quantification. We recovered approximately 55% of the bacterial cells, whereas the isolation efficiency from blood was less than 10% (SI Figure 3). This difference indicates that the interaction between *S. aureus* and blood cells is stronger than the fluid drag, transporting the bacteria into the sediment .

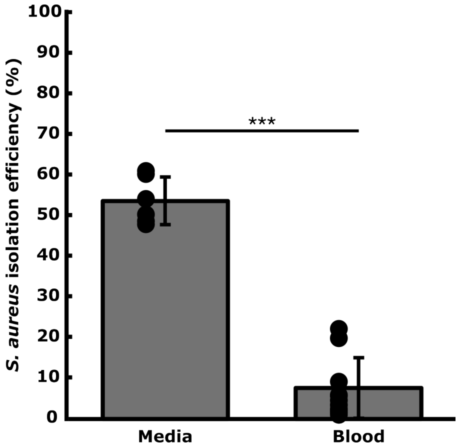

SI Figure 3: **Isolation efficiency of *S. aureus* from blood and from the same density control liquid in smart centrifugation.** *** indicates significance levels p $\leq$ 0.001. n=6 samples were tested for media, and n=10 blood samples were tested. The figure was created with Microsoft Excel and edited using the open-source software Inkscape.

Distribution of bacteria in the supernatant and pellet after smart centrifugation

We investigated the distribution of *E. coli* in the pellet and the supernatant after smart centrifugation. After the centrifugation step, the supernatant was removed and the pellet was resuspended in 2 ml of BCM to make plate counting more accurate as a very high number of interfering blood cells can decrease the accuracy of plate counting. We recovered almost all (> 90%) of the spiked bacteria. 30% of the bacteria were found in the pellet.

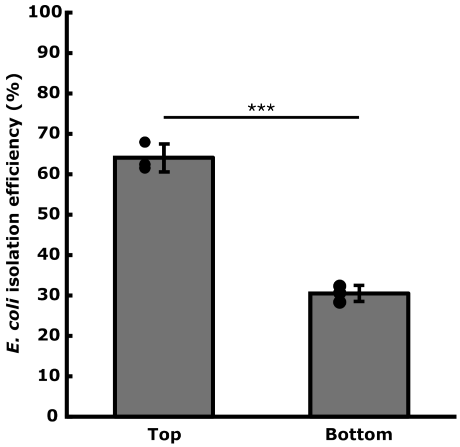

SI Figure 4: **Distribution of bacteria in supernatant and pellet after Smart centrifugation.** *** indicates significance levels p $\leq$ 0.001. n=3 blood samples were tested per condition. The figure was created with Microsoft Excel and edited using the open-source software Inkscape.

Volume reduction with and without diluted percoll as sedimentation interface

Placing a bottom layer of diluted percoll (1:2; percoll:BCM) prevents the aggregation of cells and lysate, resulting in more stable and reproducible flow rates. SI Figure 5 shows how the filtrate and retentate flow in the presence or absence of high-density media (n=5). The use of high-density media in the volume reduction step resulted in better resuspension of the lysate and cells, thus ensuring smoother and more reproducible flow. Additionally, we tested how the presence of a density media at the bottom affected the bacteria isolation efficiency during volume reduction. The isolation efficiency with percoll was found 15% higher than without the percoll, although this difference was not (SI Figure 6).

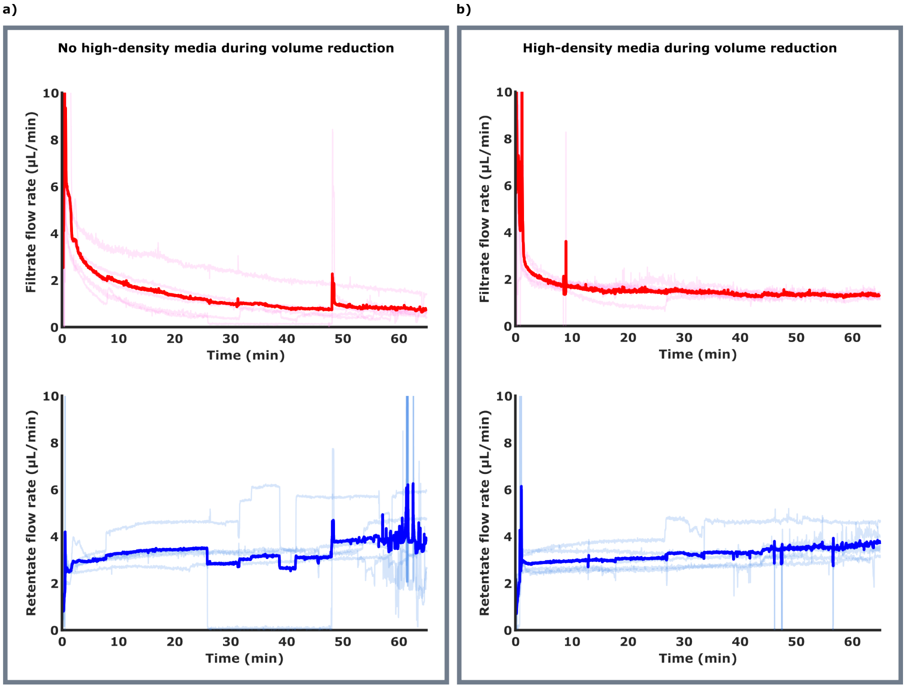

SI Figure 5: Filtrate and retentate flows are smoother and more reproducible when using high-density media in the volume reduction step. n=5 blood samples were tested per condition, 10 samples in total. The figure was created with MATLAB R2021 and edited using the open-source software Inkscape.

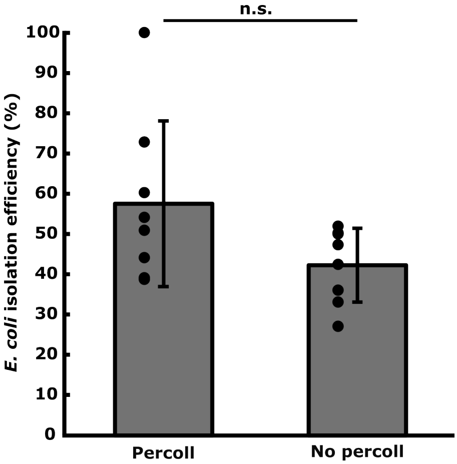

SI Figure 6: Comparison of *E. coli* isolation efficiency after volume reduction with and without diluted percoll at the bottom. ns means not significant (n=8 for each condition). The figure was created with Microsoft Excel and edited using the open-source software Inkscape.

Filtrate flow rate and overall trapping efficiency

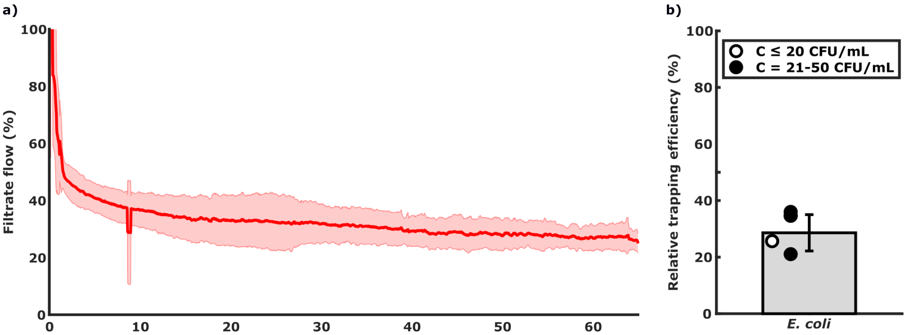

SI Figure 7: **a) Filtrate flow rate** for *E. coli*-spiked sample, where the solid line is the mean flow through the microtraps relative to the sample input flow and the shaded area indicates one standard deviation (n=5). **b) Trapping efficiency** for *E. coli*-spiked sample, which is the number of bacteria-containing traps after 70 min relative to the colony-forming units in the sample after smart centrifugation (bar height is mean, error bar is sd) (n=5). The figure was created with MATLAB R2021 and edited using the open-source software Inkscape.

Overall detection rates

The measurements plotted in SI Figure 8 are the same as in Figure 3a), but now shown as detection rate, meaning the number of bacteria-containing traps after 70 min on-chip sample flow relative to the number of colony-forming units spiked in the blood.

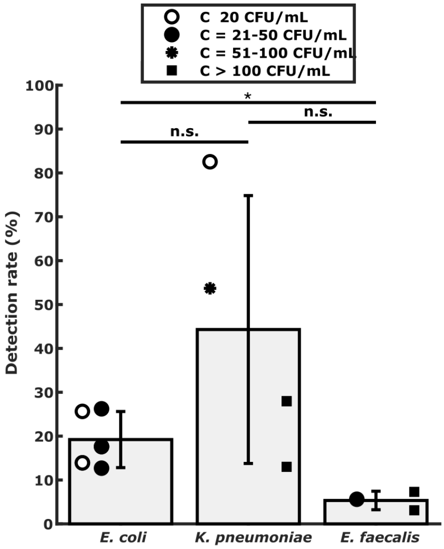

SI Figure 8: Detection rate, meaning the number of bacteria-containing traps after 70 min on-chip sample flow relative to the number of colony-forming units spiked in the blood. ns and * indicate significance levels $p > 0.05$ and $p \leq 0.05$, respectively (n=12). The figure was created with MATLAB R2021 and edited using the open-source software Inkscape.

Optimization of smart centrifugation parameters

The optimization began by layering undiluted blood over a volume of Lymphoprep in a blood collection tube (Vacutainer® blood collection tubes), which has a smaller diameter than a standard 15 mL centrifuge tube. Initially, we used Lymphoprep alone (density 1.077 g/ml), and blood cell removal was assessed visually by observing the color of the supernatant. We then optimized the volume of Lymphoprep using 0.5 mL and 1 mL, along with 4 mL of undiluted blood (results shown in SI Figure 9).

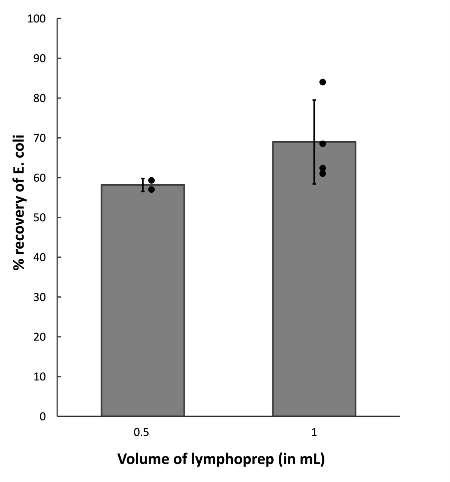

SI Figure 9: Optimization of the volume of density medium (Lymphoprep). Recovery of E. coli using two different volumes of density medium—0.5 mL and 1 mL—placed at the bottom of the blood tube, above which 4 mL of undiluted blood was layered and centrifuged. n=6 blood samples were tested. The figure was created with Microsoft Excel.

In this setup, while most red blood cells (RBCs) were effectively removed from the supernatant, white blood cells (WBCs, density range 1.057–1.092 g/ml) and platelets (1.072–1.077 g/ml) remained due to their lower densities. To facilitate their sedimentation, we decreased the density of the separation medium by mixing Lymphoprep with BCM to achieve a final density of 1.051 g/ml, which is below the density of the lightest WBCs. We also switched to a standard 15 mL centrifuge tube, which has a larger cross-sectional area, aiding in more efficient separation.

We empirically optimized the total sample volume in the 15 mL tubes as well. Volumes above 5 mL were found to cause revortexing of the sediment upon centrifuge deceleration, leading to remixing of blood cells. Therefore, a total volume of 4 mL (3 mL diluted blood + 1 mL density medium) was selected as the optimal condition.

Furthermore, to ensure that the blood sample could be properly layered on top of the separation medium, we reduced its density by diluting whole blood in a 3:1 ratio with BCM. This resulted in a sample density of approximately 1.040 g/ml, enabling layering and optimal separation. In addition to the optimized combination described above, we tested other ratios of Lymphoprep:BCM and blood:BCM while keeping the total volume constant at 4 mL. The results from these alternative combinations, along with their respective ratios, are shown in the SI Figure 10. However, we selected the value described in the manuscript, as it yielded the best results.

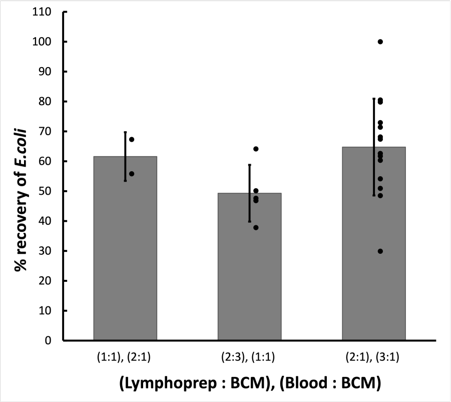

SI Figure 10: : Bacterial recovery with different dilution combinations of Lymphoprep and blood. Recovery of E. coli using three different combinations of Lymphoprep:BCM and blood:BCM mixing ratios, while keeping the volumes of the density medium and blood mixture fixed at 1 mL and 3 mL, respectively. The last histogram performed the best and was therefore used in the manuscript. n=21 blood samples were tested. The figure was created with Microsoft Excel.

Comparison table of different bacterial isolation methods

SI Table 3: Key performance parameters of smart centrifugation and other separation methods for *E.coli*.

	Bacteria isolation efficiency	RBC removal efficiency	Blood processed (ml)	Time (min)	Throughput (µl/min)	Minimum concentration C detected (CFU/ml)	Volume concentration (Vf / Vi)	Requires non-standard equipment
**Smart centrifugation**	65%	99.98%	2.25	5	450	9	1.1	No
Filter based centrifugation [3]	26-40%	99.4%	1	60	16.7	10	18	No
Compact Disk [2]	40%	94%	7	1	7000	10	0.6	Yes
Dextran sedimentation [4]	50-60%	>90%	10	30	333	10-100	0.4	No
Elastoinertial Separation [5]	>65%	100%	1	40	25	40 CFU	1.5	Yes
Inertial lift forces [6]	>80%	90%	0.15	4	37.5	$>10^5$	50	Yes
Dielectrophoreis [7]	87.2%	100%	0.05	75	0.67	10000	0.01	Yes
SAW [8]	88%	99.6%	0.012	600	0.02	$4.4*10^7$	25	Yes
Acoustophoresis [9]	75%	99.99%	1	50	20	$5*10^8$	3	Yes
Magnetic Bead Separation [10]	>80%	>99.99%	5	60	83.3	1	0.2	Yes

Data cleaning and label adjustments

A total of six positions from three experiments were discarded (due to the cropping preprocessing made an error (the barcodes were not clearly visible due to illumination and focus issues). After performing the initial labeling using the developed labeling software, we trained networks and inspected network misclassifications. From this, we corrected the labels of 159 out of 532,782 negative frames to positive and 3 out of 3,231 positive frames to negative in the test set. Furthermore, we corrected the labels of 502 out of 268,105 negative frames to positive and 231 out of 179,811 positive frames to negative in the training set. Only clearly erroneous labels were changed, where we could observe growth, or the traps were completely empty. Since only a small fraction of frames in the test set were corrected, they could not have significantly inflated the metrics due to bias. The labels of ambiguous traps were left unchanged.

Video classification

The detection was posed as a video classification problem, where one test instance contained 1-8 frames corresponding to the evaluation time of 0-70 minutes. The number of positive traps changes over time as cells get trapped in previously empty traps, as seen in SI Table 6. Because of the low concentration of bacteria, only 1% of the traps are positive at 70 minutes.

The training set consisted of 57,608 time-lapses from eight experiments, containing 5-11 frames, outlined in SI Table 4, and the test set consisted of 66,869 time-lapses also from eight experiments show in SI Table 5.

**Experiment**	**Empty**	**Bacteria**	**Total**
E. coli (EXP-24-CA6233) (7 frames)	7557	800	8357
E. coli (EXP-25-CA6232) (7 frames)	728	3587	4315
E. faecalis (EXP-25-CA6237) (6 frames)	3345	4870	8215
E. faecalis (EXP-25-CA6238) (8 frames)	8362	2	8364
K. pneumoniae (EXP-25-CA6234) (7 frames)	1942	6407	8349
K. pneumoniae (EXP-25-CA6235) (7 frames)	671	7712	8383
No bacteria (EXP-24-CA6229) (11 frames)	8155	0	8155
No bacteria (EXP-25-CA6236) (5 frames)	3470	0	3470
**Total**	34230	23378	57608

SI Table 4: The training set, showing individual experiments, species, number of frames, positive, negative, and total instances. A trap is shown as empty in the table if none of the frames in the time-lapse contains bacteria.

When employing the image classification models (ResNet 18, DinoV2, and EfficientNet B2), frames were concatenated horizontally to a time-lapse image, allowing us to employ image classification models for the video detection task. When testing (or training) on time-lapses with less than eight frames, the images were padded on the right to 336-pixel width, so all inputs had the

Experiment	Time step (minutes) 0	10	20	30	40	50	60	70
E. coli (EXP-25-CA6231)	9	13	13	13	14	14	14	14
E. faecalis (EXP-23-CA6223)	0	1	1	1	3	6	9	10
E. faecalis (EXP-23-CA6225)	8	33	62	90	105	124	149	168
E. faecalis (EXP-23-CA6226)	2	3	8	10	10	10	10	10
K. pneumoniae (EXP-23-CA6217)	21	70	118	189	202	255	354	374
K. pneumoniae (EXP-23-CA6219)	2	5	9	18	22	37	58	63
K. pneumoniae (EXP-23-CA6222)	0	1	3	5	8	12	14	15
K. pneumoniae (EXP-23-CA6230)	6	26	45	59	64	65	67	67
**Total**	48	152	259	385	428	523	675	721

SI Table 5: The test set with the number of positive traps recorded at different time steps for all experiments. The whole test set contains 66869 traps, with 66148 being empty at 70 minutes.

Time (min)	Number of positive traps	Fraction positive
0	48	0.000718
10	152	0.002273
20	259	0.003873
30	385	0.005758
40	428	0.006401
50	523	0.007821
60	675	0.010094
70	721	0.010782

SI Table 6: The test set with the number of positive traps recorded at different time steps for all eight experiments. The whole test set contains 66869 traps, with 66148 being empty at 70 minutes.

same spatial shape (this can also be viewed as masking out later frames). The appearance of image model inputs at different time points is shown in SI Figure 11a). Furthermore, the performance of all models was tested by training and testing on downsampled images shown in 11b).

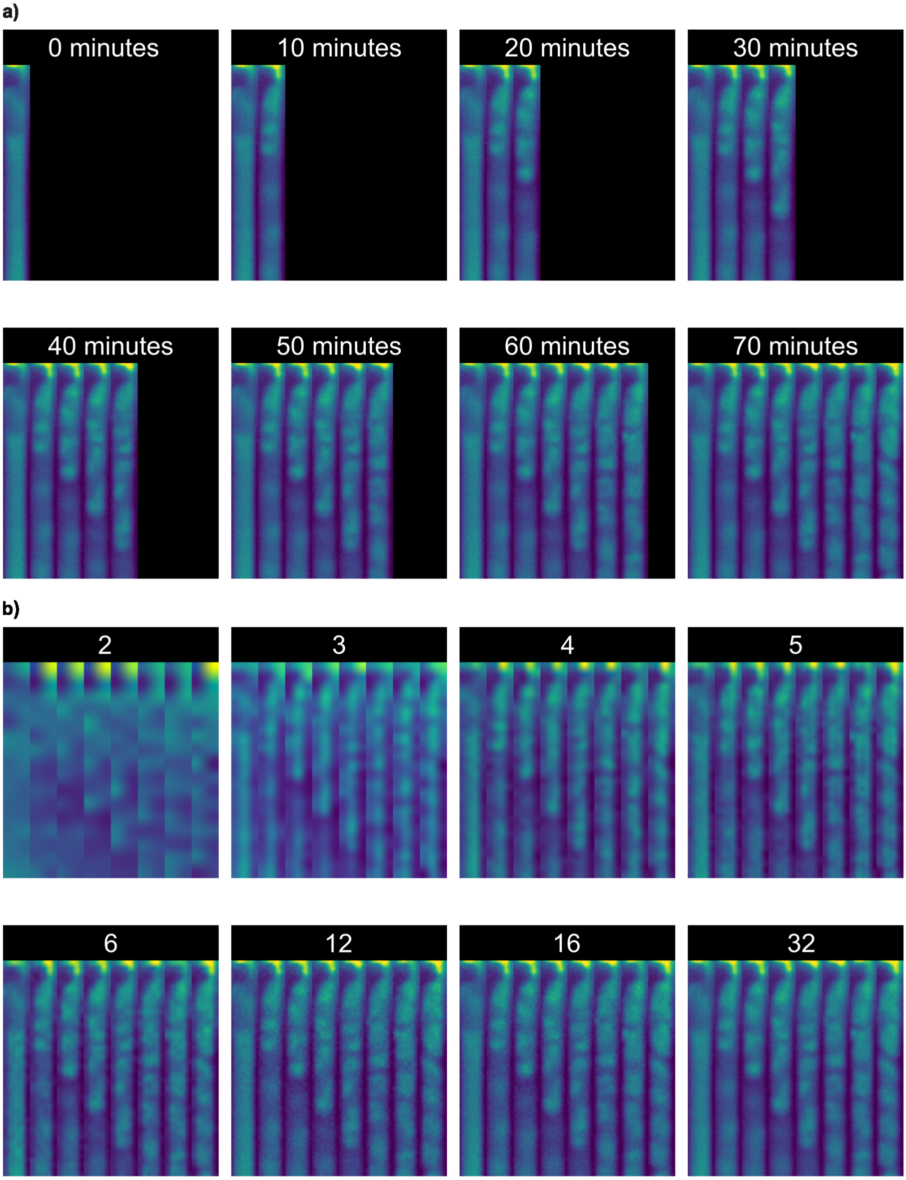

SI Figure 11: **Model input** a) Time-lapse images (336x336 pixels) of one trap processed by the image classification models ResNet 18, DinoV2, and EfficientNet B2 during the time evaluation. b) The same trap at 70 minutes imaged at progressively smaller subsampling widths, upsampled to the original size using bicubic interpolation. The figure was created using the open-source software Inkscape and the Python plotting library Matplotlib.

Although DinoV2 achieved the best results after 70 minutes, the other models had a slight edge in the first frames, especially the Video ResNet, shown in SI Figure 12. This may be attributed to the rather unnatural image of one 336x42 frame zero-padded on the right that was inputted to the image networks. However, in later stages, the Video ResNet has poorer generalization. The long timestep of 10 minutes between frames is perhaps not ideal to be processed by temporal convolutions (consecutive frames have too large a difference).

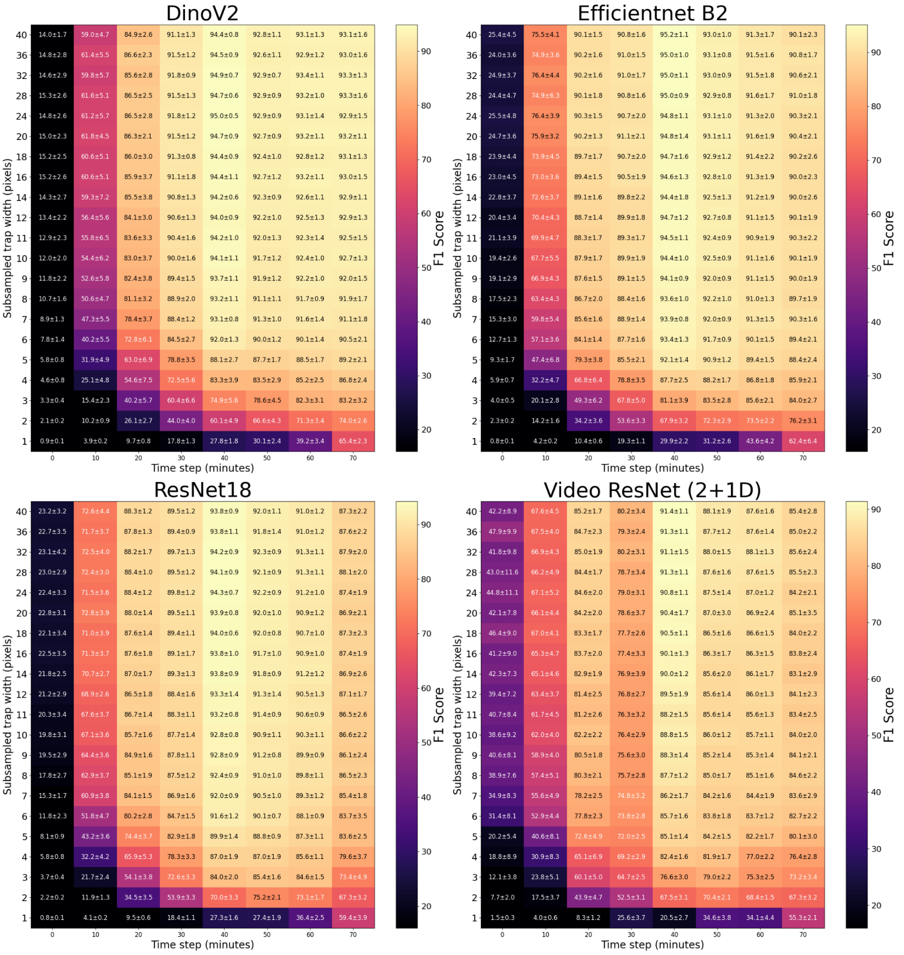

SI Figure 12: Comparison of F1 score of all models, framing the problem as video classification, subsampling, and testing on more frames. The figure was created using the open-source software Inkscape and the Python plotting library Matplotlib.

Misclassifications

The best-achieving model, DinoV2, had 84 false negatives and four false positives from the test partition at 70 minutes. The false positives and a subset of the false negatives are shown in SI Figure 13. Despite our data cleaning effort, sample a), c), d), e), and f) have the wrong label and are true negatives (the dark cell-looking objects in a), d), and e) are actually debris and lack the characteristic "gleaming" bacteria texture)

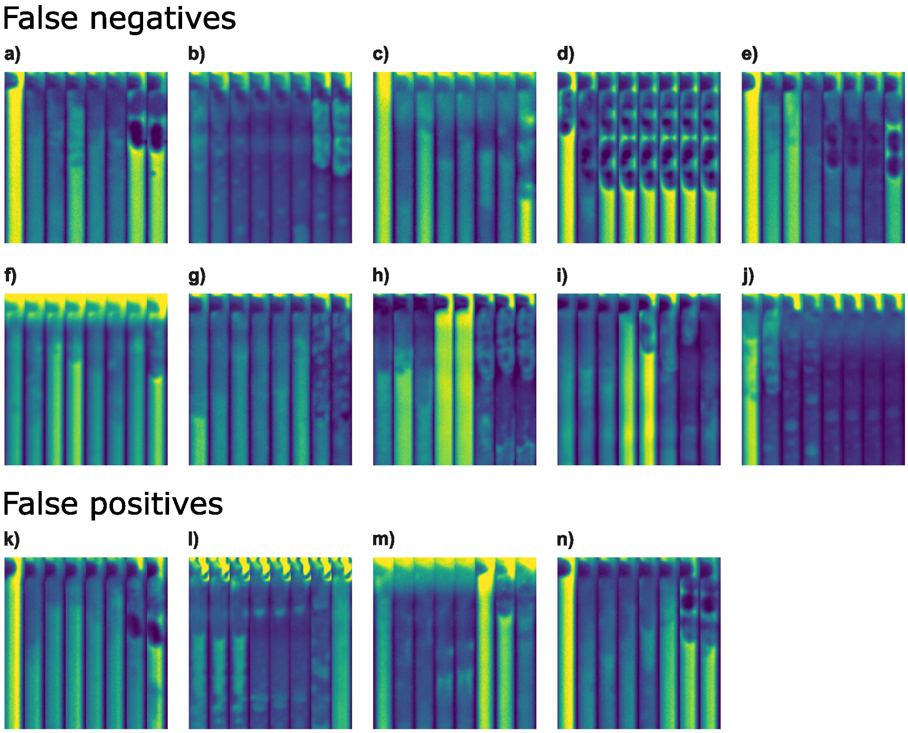

SI Figure 13: False negatives and false positives misclassified by DinoV2 at 70 minutes, full resolution. The figure was created using the open-source software Inkscape and the Python plotting library Matplotlib.

Inference times and computational complexity

The FLOPs count stated in the results section was calculated on inference shapes of 336x336 pixels for the image models and 8x336x42 pixels for Video ResNet. The inference latency of one sample on the A100 GPU was 1.410 ± 0.086ms for ResNet 18, 1.693 ± 0.051ms for EfficientNet B2, 3.085 ± 0.032ms for DinoV2, and 1.573 ± 0.046ms for Video ResNet (2+1D) (using Pytorch data

loader with 5 workers, test batch size 32). However, the inference times could be improved using software and hardware optimizations in a live setting.

Posing the problem as single-frame image classification

In our study, we posed the problem as a video classification problem, treating a time-lapse of an individual trap as one classification instance. A trap was assigned a positive label if it contained or had previously contained any cells until the evaluation time point. This problem framing aligns better with a potential clinical application. However, since we have frame-level annotations, we can treat it as a simple image classification problem and independently classify each frame, disregarding the time lapses. One classification instance is then a single frame from any trap time-lapse in any experiment and has a positive label only if it contains bacteria. Processing single-frames could be more feasible if operating on resource-constrained hardware. The smaller memory footprint allowed us to use the whole 1300x40 pixel trap images as inputs as opposed to cropping the height at 336 pixels for the video classification. The train and test sets are shown in SI Table7 and SI Table8. Analogous to the video classification, all single-frame networks were retrained 30 times with unique random seeds for reproducibility and statistics. When performing subsampling experiments, the images were upsampled back to the original size using bicubic interpolation before feeding into the networks. All single-frame networks were trained for 50 epochs using batch size 256, a learning rate of 10^{-3} for all CNN:s and 10^{-5} for DinoV2, otherwise the same settings as when training for video classification (except random frame erase).

**Experiment**	**Empty**	**Bacteria**	**Total**
E. coli (EXP-24-CA6233) (7 frames)	52467	6154	58621
E. coli (EXP-25-CA6232) (7 frames)	5089	42832	47921
E. faecalis (EXP-25-CA6237) (6 frames)	20013	29503	49516
E. faecalis (EXP-25-CA6238) (8 frames)	66928	100	67028
K. pneumoniae (EXP-25-CA6234) (7 frames)	11325	47266	58591
K. pneumoniae (EXP-25-CA6235) (7 frames)	4478	54227	58705
No bacteria (EXP-24-CA6229) (11 frames)	90184	0	90184
No bacteria (EXP-25-CA6236) (5 frames)	17350	0	17350
Total	267834	180082	447916

SI Table 7: The training set for single-frame classification, showing individual experiments, species, positive, negative, and total single-frame images.

Subsampling single-frames

We also wanted to study the performance at lower resolution for the single-frames, training, and testing on downsampled images. The results shown in SI Figure 14, a sharp perfomance drop happens in about 8-pixel width, indicating that there is plenty of room to use lower-resolution microscopy for the task.

Experiment	Empty	Bacteria	Total
E. coli (EXP-25-CA6231)	67034	144	67178
E. faecalis (EXP-23-CA6223)	66505	23	66528
E. faecalis (EXP-23-CA6225)	66413	751	67164
E. faecalis (EXP-23-CA6226)	67133	63	67196
K. pneumoniae (EXP-23-CA6217)	65164	1728	66892
K. pneumoniae (EXP-23-CA6219)	66642	214	66856
K. pneumoniae (EXP-23-CA6222)	67140	58	67198
K. pneumoniae (EXP-23-CA6230)	66595	406	67001
Total	532626	3387	536013

SI Table 8: The test set for single-frame classification, showing individual experiments, species, positive, negative, and total single-frame images.

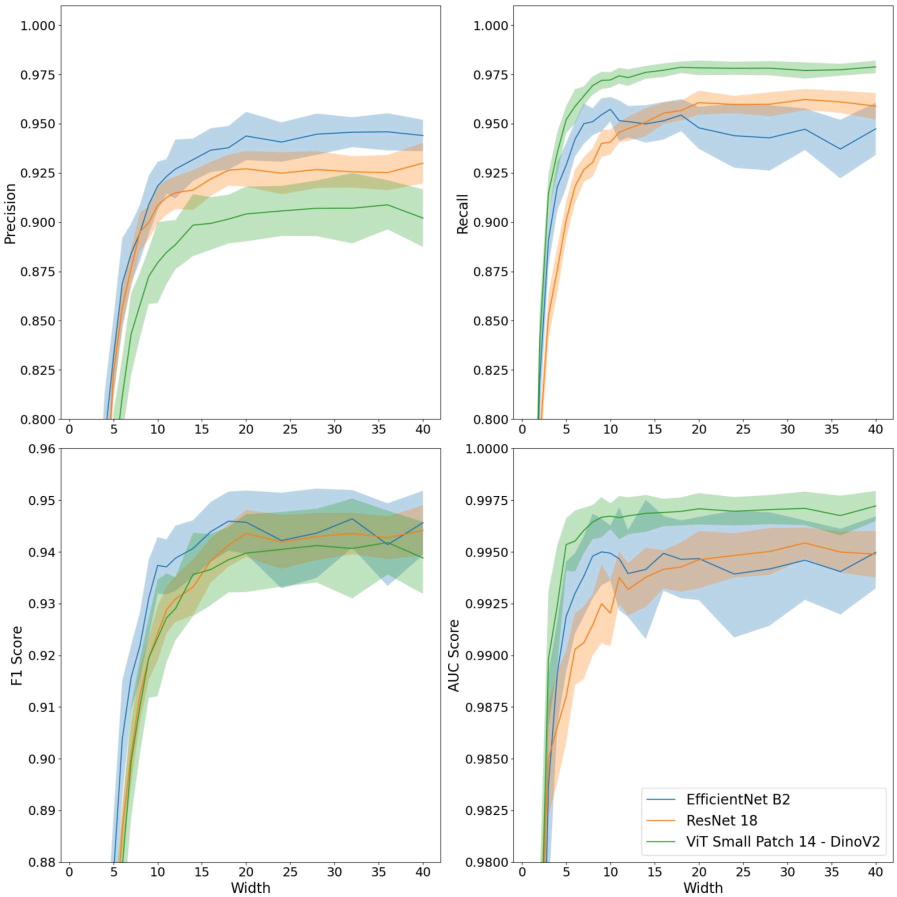

SI Figure 14: Performance metrics of all image models when processing sub-sampled images. Lines show mean, and shaded areas show standard deviations among the 30 retrainings. The figure was created using the open-source software Inkscape and the Python plotting library Matplotlib.

Images of cells at different time points

Typically, frames from earlier timesteps were more challenging to classify because the cells are significantly smaller, lack the characteristic bacteria cell texture, and are situated at the very top of the microfluidic trap. Therefore, to study the performance of the single-frame classification network at different time points, we partitioned the test set based on the time at which each frame was captured and calculated the classification performance for each subset. The interesting results are shown in SI Figure 15. For the first few frames, it is more advantageous to use single-frame models. When processing 1300x40 frames, it is easier for the network to see the top end of the bacterial column, which could be important at lower resolution. The results at 70 minutes were 95.3+-0.6 for DinoV2, 95.2+-0.6 for Efficientnet B2, and 94.7+-0.5 for ResNet 18. Note that it is problematic to compare the final performance to the video models: Hard samples where traps previously contained cells that subsequently escaped are still labeled positive for the video models at 70 minutes.

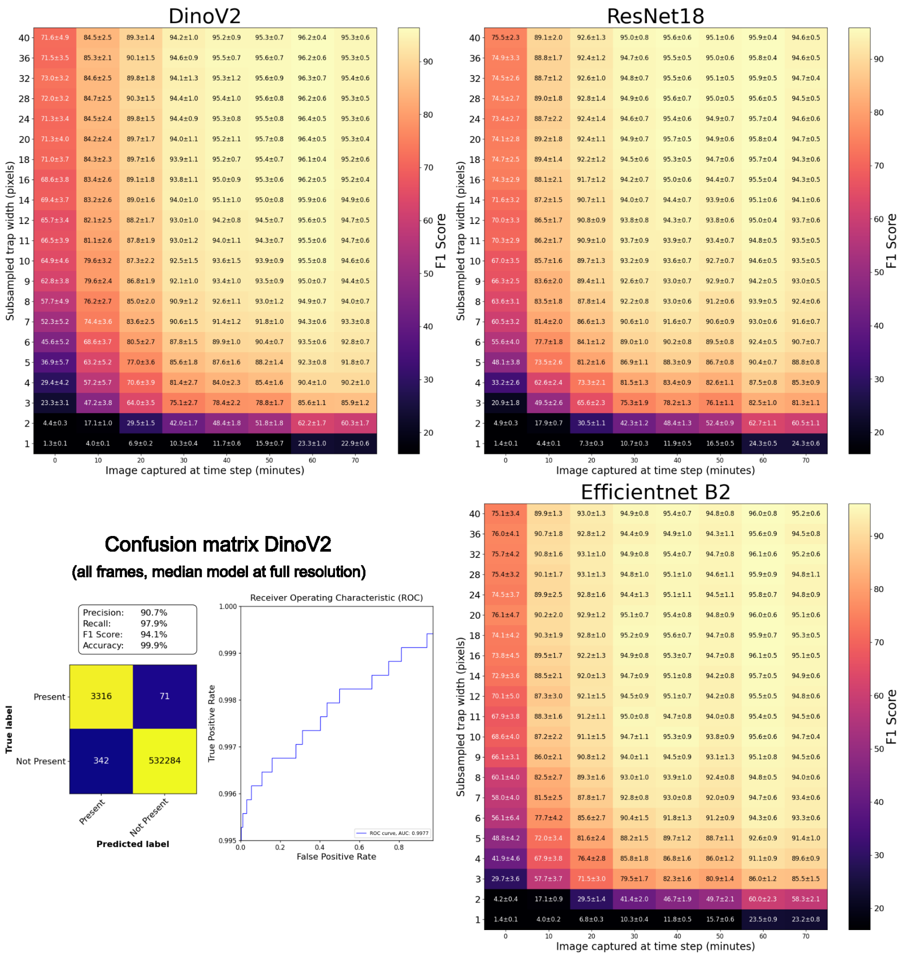

SI Figure 15: Single-frame classification results, partitioning the test set according to when a test frame was taken. The lower left shows the confusion matrix and AUC of the best model, DinoV2, testing on frames from all timesteps, showing the model with the median F1-score of the 30 retrainings. The figure was created using the open-source software Inkscape and the Python plotting library Matplotlib.

Reshaping the oblong images during single-frame classification

All our studies used networks initialized with pretrained weights, either on ImageNet, or self-supervised DinoV2. The pretraining was performed on natural images with image size 224x224 pixels, very different from our oblong

1300x40 pixel trap images. We wanted to see how reshaping affects performance, so we performed experiments split up the trap into patches of 224x40 and stacked horizontally using three strategies:

1. Reshaping and performing our usual padding to 42-pixel width, resulting in an input image of 224x256.
2. Reshaping but cropping each trap to 39 pixels and cropping the final reshaped frame 10 pixels to an image size of 224x224.
3. Performing no reshaping, using the unchanged 1300x40 pixel images.

Results are shown in SI Figure 16, each network was retrained 50 times using different random seeds. Despite ResNets and EfficientNets using the same Global Average Pooling in the final layer, EfficientNet performs better. Thus, it is better at extracting features regardless of shape. For all our experiments, we chose the first strategy, thus reshaping the single-frame images 224x256 before feeding into the network.

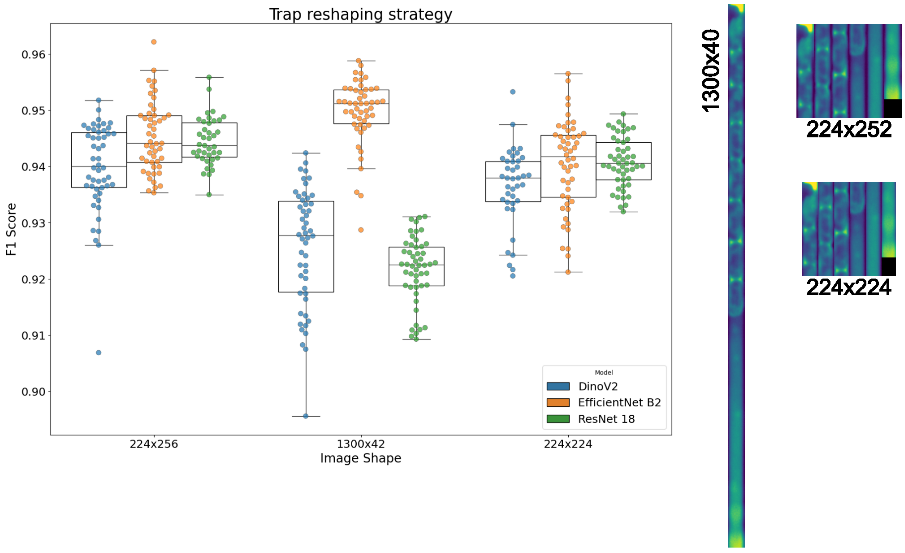

SI Figure 16: Retraining networks using different reshaping strategies. The figure was created using the open-source software Inkscape and the Python plotting library Matplotlib.

Negative control tests

We performed experiments (n=3) using non-spiked sterile blood to assess false positive rates and evaluate the specificity of the detection, while ruling out possible manual mislabelings. One instance of DinoV2, EfficientNet B2, and ResNet18 was used for the evaluation, both in using video detection mode (using horizontally concatenated frames) and using single-frame classification.

The results are shown in SI Figure 17, indicating that video detection performs better compared to single-frame detection, as the models using video data correctly identify the absence of growth over time. Notably, Video ResNet18 produced only one false positive at 0 minutes (accessing only one frame). Each experiment contains 8000 traps. For context, the 24 false positives produced by EfficientNet B2 at frame 1 (0 minutes) in Experiment 2 correspond to the highest observed false positive rate of just 0.3%. A few reoccurring false positive misclassifications are shown in SI Figure 18.

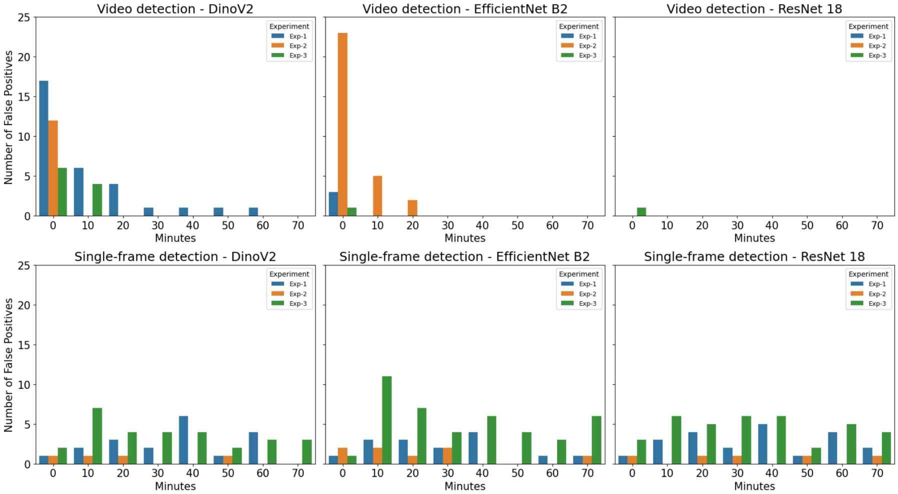

SI Figure 17: Test results, number of false positives of the models across three control experiments, each including 8000 traps. The figure was created using the open-source software Inkscape and the Python plotting library Matplotlib.

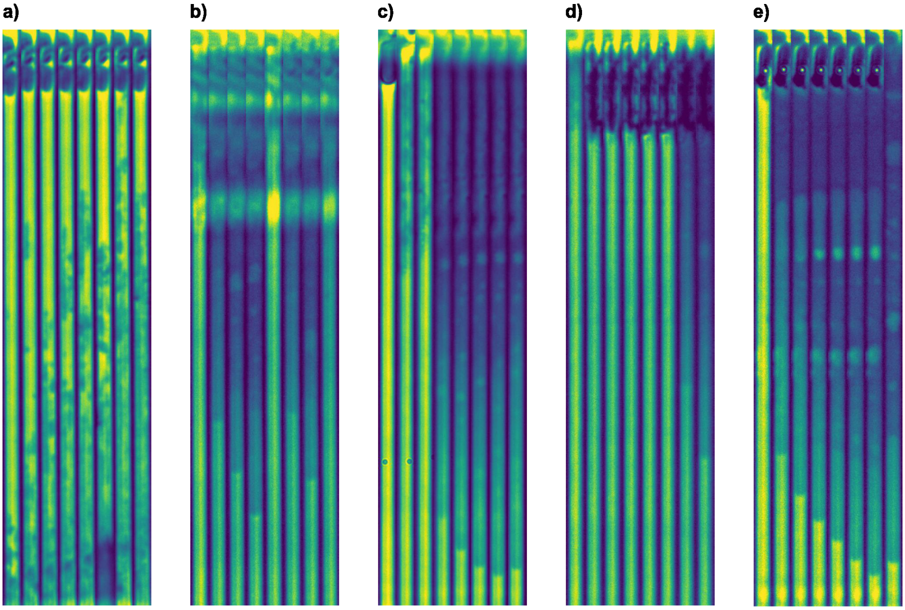

SI Figure 18: A few traps with debris classified by the video models as false positives across three empty experiments. a) (Exp-3): DinoV2 (frames 1–2), EfficientNet B2 (frame 1), ResNet-18 (frame 1). b) (Exp-1): DinoV2 (frames 1–3). c) (Exp-2): DinoV2 (frame 1), EfficientNet B2 (frame 1). d) (Exp-1): DinoV2 (frames 4–6). E (Exp-1): EfficientNet B2 (frame 1). The figure was created using the open-source software Inkscape and the Python plotting library Matplotlib.

Image preprocessing and cropping out traps

An image processing pipeline was further improved from [11] to rotate, crop out, and stabilize the individual traps in the microfluidic chip. The preprocessing code is available in the released software package [12] and was developed using OpenCV[13], Numpy[14], Scipy[15], and MatPlotLib[16]. The pipeline consists of the following steps:

1. Rotate the frames by a few degrees to align the chip structure horizontally.
2. Project the image on the horizontal axis to find the location of the traps and barcodes in each frame.
3. Determine which peaks correspond to barcodes and assign a location ID to each trap.
4. Identify the location of the physical stop protruding out from the trap wall and determine where to crop the traps along the vertical axis.
5. Crop and stack the 1300x40 pixel trap images, and perform fine-grained stabilization using StackReg.[17]

For the rotation step, we first applied Gaussian blur to the images, then adaptive thresholding, calculating the contours, and finally projecting the binary image on the horizontal and vertical axes. When the chip structure was perfectly horizontal, the sums of the projections were at maximum values (trap walls aligned vertically). A grid search was performed with progressively smaller steps to find the optimal rotation angle.

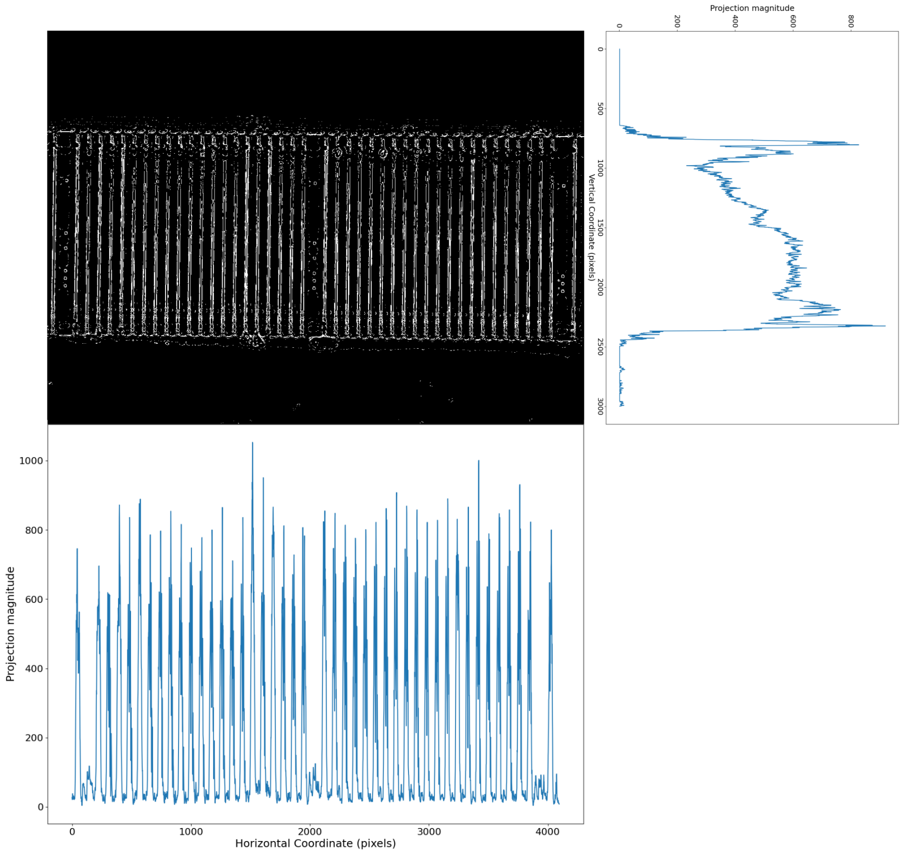

SI Figure 19: Projections on the horizontal and vertical axes were used to find the optimal rotation angle. The figure was created using the open-source software Inkscape and the Python plotting library Matplotlib.

Then, the raw images were projected on the horizontal axis, followed by 1D Gaussian blur, revealing the horizontal location of the traps 20a) The barcodes were also visible as peaks in the projection. The traps were cropped along the vertical axis by a 20-pixel displacement on each side of a peak. A barcode score was assigned to the three leftmost, rightmost, and middle crop-outs to determine the most likely barcode locations. This score was based on whether

there were any peaks when projecting the cropped-out images on the vertical axis, the 75th percentile pixel value (barcodes images had darker pixels on average), and a few other metrics. In practice, finding a robust metric was challenging: a trap could be very dark with bright peaks if filled with cocci cells, and conversely, a barcode could be bright if it contained many barcode dots. By knowing the barcode locations, we could assign a trap ID to each crop out and pair them across the different frames in the time-lapse. This was necessary as the raw images often exhibited significant drift during the time-lapse.

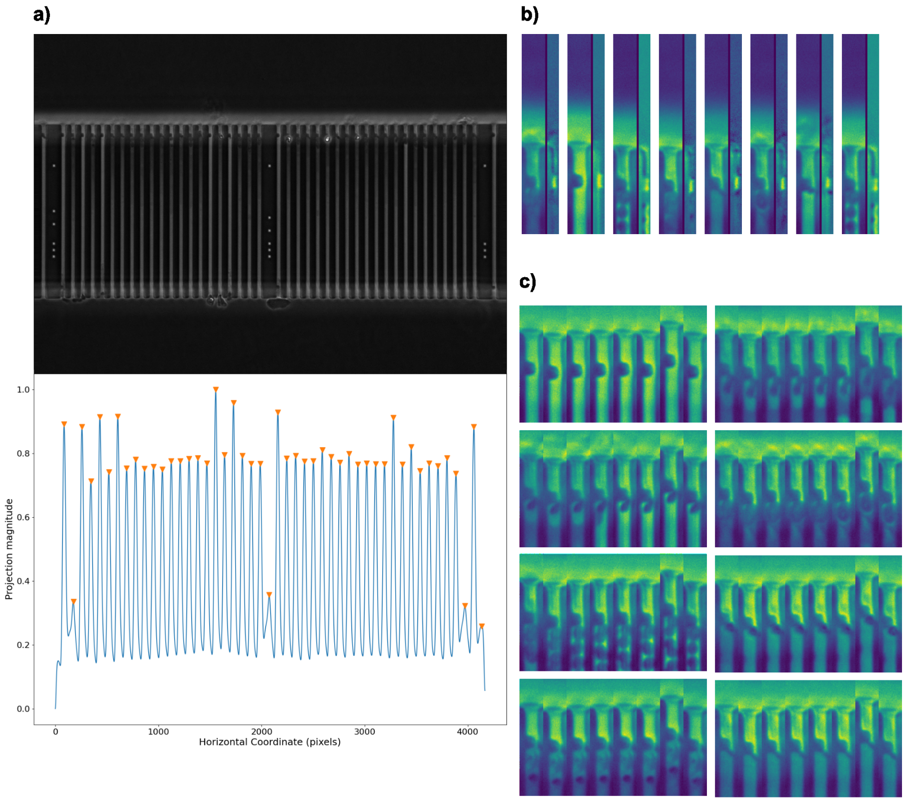

SI Figure 20: a)Projecting the traps on the horizontal axis followed by applying 1D Gaussian 1D kernel, with the peaks showing the trap locations. b) Using the asymmetry introduced by the physical stop protruding out from the side of the wall to find the location to crop horizontally. Images of eight separate traps are shown, horizontally concatenated with a dark border and the calculated image difference, revealing the stop as a bright spot. c) The fine-grained stabilization of the cropped-out phase-contrast time-lapse stack was calculated using the movement of the physical stop-stop (300x40 pixel crop). The images show eight different traps and the corresponding frames in the time-lapse horizontally concatenated. There was a larger vertical shift in the seventh frame, later corrected with the fine-grained stabilization. The figure was created using the open-source software Inkscape and the Python plotting library Matplotlib.

The vertically cropped trap images were then cropped on the horizontal axis right at the location of the physical stop. The stop introduced an asymmetry in the image, and when calculating the image difference of the left and the

horizontally flipped right image-half (cropped vertically at the center) revealed the stop as a bright peak 20B, pseudocode outlined below:

$$
\begin{aligned}
&\text{Let } \mathbf{IM}_{\text{trap}} \in \mathbb{R}^{1300\times 40}\\
&\mathbf{IM}_{\text{left}} = \mathbf{IM}_{\text{trap}}[:, 1:20],\\
&\mathbf{IM}_{\text{right}} = \mathbf{IM}_{\text{trap}}[:, 21:40],\\
&\mathbf{IM}_{\text{diff}} = \text{FlipLeftRight}(\mathbf{IM}_{\text{right}}) - \mathbf{IM}_{\text{left}}.
\end{aligned}
$$

After cropping, the time-lapse of each trap was fine-grained stabilized using StackReg[17] using only translation. The transformation matrices were calculated using a small 300x40 pixel cropout containing the physical stop, a few samples are shown in 20c) Finally, the color map Vidiris[18] was applied to the single-channel phase-contrast microscopy images to be easily viewed by a human labeler.

Tabled experimental measurements and their results

The tables below contain the detailed experimental description and raw data of plate counts for all the experiments included in this study.

Expt. id is the unique identification number of the experiment.

Expt. type is the type of experiment, meaning either coventional centrifugation, smart centrifugation, volume reduction and detection in microfluidics trap, refer Methods.

Control plate count is the bacteria count after plating 100 µl of the spiking solution used to spike the blood (n=3). Columns 2-4 show the number of colonies on the plate. The last column shows the mean count.

Expt. plate count is the bacteria count after plating 100 µl of recovered solution after a centrifugation steps. Columns 2-4 show the number of colonies on the plate. The last column shows the mean count.

Volume recovered (ml) is the volume recovered after centrifugation.

Isolation efficiency (%) is the efficiency of bacterial separation of the experiment, meaning the number of bacteria recovered relative to the number of bacteria initially spiked.

Concentration in blood (CFU/ml) is the concentration of bacteria in the spiked blood, C.

Bacteria (t60), (t70) and (tend) are the number of bacteria trapped in the microtraps after 60 min on-chip processing, 70 min on-chip processing, and at the end of the experiment.

Overall assay performance is the number of positive microtraps detected, N, versus the bacterial concentration in the blood sample, C.

SI Table 9: Raw data of plate counts of all experiments with *E. coli.*

				Mean
Expt. id (Date / Name)	2022-12-02			
Expt. type	Conventional centrifugation			
Control plate count	42	71	69	60.7
Expt. plate count	23	50	55	42.7
Volume recovered (ml)	2.0			
Isolation efficiency (%)	39.7			
Concentration in blood (CFU/ml)	537.7			
Expt. id (Date / Name)	2022-12-05			
Expt. type	Conventional centrifugation			
Control plate count	48	32	29	36
Expt. plate count	27	14	26	22.3
Volume recovered (ml)	1.9			
Isolation efficiency (%)	32.9			

Concentration in blood (CFU/ml)	322.0			
Expt. id (Date / Name)	2022-12-07			
Expt. type	Conventional centrifugation			
Control plate count	45	32		38.5
Expt. plate count	12	21	26	19.7
Volume recovered (ml)	1.9			
Isolation efficiency (%)	26.7			
Concentration in blood (CFU/ml)	295.8			
Expt. id (Date / Name)	2023-03-02 (a)			
Expt. type	Smart centrifugation			
Control plate count	58	52	43	51
Expt. plate count	42	36	39	39
Volume recovered (ml)	2.8			
Isolation efficiency (%)	71.4			
Concentration in blood (CFU/ml)	680			
Expt. id (Date / Name)	2023-03-02 (b)			
Expt. type	Smart centrifugation			
Control plate count	58	52	43	51
Expt. plate count	41	30		35.5
Volume recovered (ml)	2.7			
Isolation efficiency (%)	62.6			
Concentration in blood (CFU/ml)	680			
Expt. id (Date / Name)	2023-03-02 (c)			
Expt. type	Smart centrifugation			
Control plate count	75	53	70	66
Expt. plate count	48	42		45
Volume recovered (ml)	3.0			
Isolation efficiency (%)	67.4			
Concentration in blood (CFU/ml)	880			
Expt. id (Date / Name)	2023-07-05 (a)			
Expt. type	Smart centrifugation			

Control plate count	161	143	158	154.0
Expt. plate count	81	75	78	78.0
Volume recovered (ml)	3.1			
Isolation efficiency (%)	79.8			
Concentration in blood (CFU/ml)	1346			
Expt. id (Date / Name)	2023-07-05 (b)			
Expt. type	Smart centrifugation			
Control plate count	161	143	158	154.0
Expt. plate count	75	64	97	78.7
Volume recovered (ml)	3.1			
Isolation efficiency (%)	80.5			
Concentration in blood (CFU/ml)	1346			
Expt. id (Date / Name)	EC Chip Trial 3			
Expt. type	Smart centrifugation + Microfluidic detection			
Control plate count	101	108	99	102.7
Expt. plate count	10	9	4	7.7
Volume recovered (ml)	2.5			
Isolation efficiency (%)	29.9			
Concentration in blood (CFU/ml)	51.0			
Bacteria (t60)	3			
Bacteria (tend)	5			
Overall assay performance (%)	17.4			
Expt. id (Date / Name)	EC Chip Trial 1			
Expt. type	Smart centrifugation + Microfluidic detection			
Control plate count	82	71	74	75.7
Expt. plate count	11	9	6	8.7
Volume recovered (ml)	2.5			
Isolation efficiency (%)	50.9			
Concentration in blood (CFU/ml)	38			
Bacteria (t60)	14			
Bacteria (tend)	19			

Overall assay performance (%)	43.8			
Expt. id (Date / Name)	EC Chip Trial 2			
Expt. type	Smart centrifugation + Microfluidic detection			
Control plate count	36	42	48	42.0
Expt. plate count	7	7	5	6.3
Volume recovered (ml)	2.5			
Isolation efficiency (%)	60.3			
Concentration in blood (CFU/ml)	21			
Bacteria (t60)	4			
Bacteria (tend)	6			
Overall assay performance (%)	21.0			
Expt. id (Date / Name)	EC Chip Trial 6			
Expt. type	Smart centrifugation + Microfluidic detection			
Control plate count	35	50	37	40.7
Expt. plate count	9	8	3	6.7
Volume recovered (ml)	2.5			
Isolation efficiency (%)	72.9			
Concentration in blood (CFU/ml)	20			
Bacteria (t60)	9			
Bacteria (tend)	24			
Overall assay performance (%)	52.5			
Expt. id (Date / Name)	EC Chip Trial 5			
Expt. type	Smart centrifugation + Microfluidic detection			
Control plate count	38	35	42	38.3
Expt. plate count	6	6	2	4.7
Volume recovered (ml)	2.5			
Isolation efficiency (%)	54.1			
Concentration in blood (CFU/ml)	19			

Bacteria (t60)	6			
Bacteria (tend)	8			
Overall assay performance (%)	18.6			
Expt. id (Date / Name)	EC Chip Trial 4			
Expt. type	Smart centrifugation + Microfluidic detection			
Control plate count	17	23	12	17.3
Expt. plate count	6	4	3	4.3
Volume recovered (ml)	2.5			
Isolation efficiency (%)	100.0			
Concentration in blood (CFU/ml)	9.0			
Bacteria (t60)	5.0			
Bacteria (tend)	8.0			
Overall assay performance (%)	41.0			
Expt. id (Date / Name)	2024-02-22 (a)			
Expt.desciption	Smart centrifugation + Standard Up-concentration			
Control plate count	128	126	145	133.0
Expt. plate count	114	137	119	123.3
Volume recovered (ml)	0.6			
Isolation efficiency (%)	44.2			
Concentration in blood (CFU/ml)	682			
Expt. id (Date / Name)	2024-02-22 (b)			
Expt.desciption	Smart centrifugation + Standard Up-concentration			
Control plate count	128	126	145	133.0
Expt. plate count	120	100	104	108.0
Volume recovered (ml)	0.6			
Isolation efficiency (%)	38.7			
Concentration in blood (CFU/ml)	682			
Expt. id (Date / Name)	2024-02-22 (c)			

Expt.desciption	Smart centrifugation + Standard Up-concentration			
Control plate count	128	126	145	133.0
Expt. plate count	120	105	103	109.3
Volume recovered (ml)	0.6			
Isolation efficiency (%)	44.2			
Concentration in blood (CFU/ml)	682			
Expt. id (Date / Name)	2024-02-22 (d)			
Expt.desciption	Smart centrifugation + Standard Up-concentration without percoll			
Control plate count	128	126	145	133.0
Expt. plate count	71	85	71	75.7
Volume recovered (ml)	0.6			
Isolation efficiency (%)	27.1			
Concentration in blood (CFU/ml)	682			
Expt. id (Date / Name)	2024-02-22 (e)			
Expt.desciption	Smart centrifugation + Standard Up-concentration without percoll			
Control plate count	128	126	145	133
Expt. plate count	99	81	97	92.3
Volume recovered (ml)	0.6			
Isolation efficiency (%)	33.1			
Concentration in blood (CFU/ml)	682.0			
Expt. id (Date / Name)	2024-02-22 (f)			
Expt.desciption	Smart centrifugation + Standard Up-concentration without percoll			
Control plate count	128	126	145	133.0
Expt. plate count	101	107	94	100.7
Volume recovered (ml)	0.6			
Isolation efficiency (%)	36.1			

Concentration in blood (CFU/ml)	682			
Expt. id (Date / Name)	2024-02-26 (a)			
Expt.desciption	Smart centrifugation + Standard Up-concentration without percoll			
Control plate count	134	136	133	134.3
Expt. plate count	209	229	202	213.3
Volume recovered (ml)	0.5			
Isolation efficiency (%)	50.0			
Concentration in blood (CFU/ml)	852.9			
Expt. id (Date / Name)	2024-02-26 (b)			
Expt.desciption	Smart centrifugation + Standard Up-concentration without percoll			
Control plate count	134	136	133	134.3
Expt. plate count	194	199	212	201.7
Volume recovered (ml)	0.5			
Isolation efficiency (%)	47.3			
Concentration in blood (CFU/ml)	852.9			
Expt. id (Date / Name)	2024-02-26 (c)			
Expt.desciption	Smart centrifugation + Standard Up-concentration without percoll			
Control plate count	134	136	133	134.3
Expt. plate count	159	195	190	181.3
Volume recovered (ml)	0.5			
Isolation efficiency (%)	42.5			
Concentration in blood (CFU/ml)	852.9			
Expt. id (Date / Name)	2024-02-26 (d)			
Expt.desciption	Smart centrifugation + Standard Up-concentration without percoll			

Control plate count	134	136	133	134.3
Expt. plate count	198	231	235	221.3
Volume recovered (ml)	0.5			
Isolation efficiency (%)	51.9			
Concentration in blood (CFU/ml)	852.9			
Expt. id (Date / Name)	2024-02-26 (e)			
Expt.desciption	Smart centrifugation + Standard Up-concentration without percoll			
Control plate count	134	136	133	134.3
Expt. plate count	222	207	215	214.7
Volume recovered (ml)	0.5			
Isolation efficiency (%)	50.3			
Concentration in blood (CFU/ml)	852.9			
Expt. id (Date / Name)	2024-02-20 (a)			
Expt.desciption	Smart centrifugation			
Control plate count	112	122	102	112.0
Expt. plate count	34	39		36.5
Volume recovered (ml)	2.6			
Isolation efficiency (%)	62.5			
Concentration in blood (CFU/ml)	933.3			
Expt. id (Date / Name)	2024-02-20 (b)			
Expt.desciption	Smart centrifugation			
Control plate count	112	122	102	112.0
Expt. plate count	41	31		36
Volume recovered (ml)	2.6			
Isolation efficiency (%)	61.7			
Concentration in blood (CFU/ml)	933.3			
Expt. id (Date / Name)	2024-02-22 (g)			
Expt.desciption	Smart centrifugation			
Control plate count	128	126	145	133.0
Expt. plate count	37	40	35	37.3
Volume recovered (ml)	2.8			
Isolation efficiency (%)	68.1			

Concentration in blood (CFU/ml)	682			

SI Table 10: Raw data of plate counts of all experiments with *K. pneumoniae*.

				Mean
Expt. id (Date / Name)	2023-09-22 (a)			
Expt. type	Smart centrifugation			
Control plate count	131			131.0
Expt. plate count	123	116	139	126.0
Volume recovered (ml)	2.7			
Isolation efficiency (%)	95.2			
Concentration in blood (CFU/ml)	1587.8			
Expt. id (Date / Name)	2023-09-22 (b)			
Expt. type	Smart centrifugation			
Control plate count	131			131.0
Expt. plate count	118	126	110	118.0
Volume recovered (ml)	2.7			
Isolation efficiency (%)	89.2			
Concentration in blood (CFU/ml)	1587.8			
Expt. id (Date / Name)	2023-09-22 (c)			
Expt. type	Smart centrifugation			
Control plate count	131			131.0
Expt. plate count	21	12	11	14.7
Volume recovered (ml)	2.6			
Isolation efficiency (%)	98.0			
Concentration in blood (CFU/ml)	172.9			
Expt. id (Date / Name)	2023-09-22 (d)			
Expt. type	Smart centrifugation			
Control plate count	131			131.0
Expt. plate count	1	0	3	1.3
Volume recovered (ml)	2.6			
Isolation efficiency (%)	97.0			
Concentration in blood (CFU/ml)	15.8			
Expt. id (Date / Name)	2023-09-22 (e)			
Expt. type	Smart centrifugation			
Control plate count	131			131.0
Expt. plate count	1	2	2	1.7
Volume recovered (ml)	2.7			

Isolation efficiency (%)	125.9			
Concentration in blood (CFU/ml)	15.8			
Expt. id (Date / Name)	2023-09-22 (f)			
Expt. type	Smart centrifugation			
Control plate count	131			131.0
Expt. plate count	1	0	3	1.3
Volume recovered (ml)	2.6			
Isolation efficiency (%)	97.0			
Concentration in blood (CFU/ml)	15.9			
Expt. id (Date / Name)	2023-09-22 (g)			
Expt. type	Smart centrifugation			
Control plate count	131			131.0
Expt. plate count	1	2	2	1.7
Volume recovered (ml)	2.7			
Isolation efficiency (%)	125.9			
Concentration in blood (CFU/ml)	15.9			
Expt. id (Date / Name)	2023-09-26 (a)			
Expt. type	Smart centrifugation			
Control plate count	212	231	213	218.7
Expt. plate count	187	184	189	186.7
Volume recovered (ml)	2.7			
Isolation efficiency (%)	82.9			
Concentration in blood (CFU/ml)	2650.5			
Expt. id (Date / Name)	2023-09-26 (b)			
Expt. type	Smart centrifugation			
Control plate count	212	231	213	218.7
Expt. plate count	25	18	15	19.3
Volume recovered (ml)	2.6			
Isolation efficiency (%)	84.3			
Concentration in blood (CFU/ml)	265			
Expt. id (Date / Name)	2023-09-26 (c)			
Expt. type	Smart centrifugation			
Control plate count	212	231	213	218.7

Expt. plate count	17	18	18	17.7
Volume recovered (ml)	2.6			
Isolation efficiency (%)	77.0			
Concentration in blood (CFU/ml)	265			
Expt. id (Date / Name)	2023-09-27 (a)			
Expt. type	Smart centrifugation			
Control plate count	175	186	187	182.7
Expt. plate count	26	25	19	23.3
Volume recovered (ml)	2.2			
Isolation efficiency (%)	103.0			
Concentration in blood (CFU/ml)	221.4			
Expt. id (Date / Name)	2023-09-27 (b)			
Expt. type	Smart centrifugation			
Control plate count	175	186	187	182.7
Expt. plate count	1	2	2	1.7
Volume recovered (ml)	2.7			
Isolation efficiency (%)	90.3			
Concentration in blood (CFU/ml)	22.1			
Expt. id (Date / Name)	2023-09-27 (c)			
Expt. type	Smart centrifugation			
Control plate count	175	186	187	182.7
Expt. plate count	2	1	2	1.7
Volume recovered (ml)	2.6			
Isolation efficiency (%)	86.9			
Concentration in blood (CFU/ml)	22.1			
Expt. id (Date / Name)	KP Chip Trial 6			
Expt. type	Smart centrifugation + Microfluidic detection			
Control plate count	4	4	3	3.7
Expt. plate count	13	13	16	14.0
Volume recovered (ml)	0.6			
Isolation efficiency (%)	132.7			
Concentration in blood (CFU/ml)	7			

Bacteria (t70)	12			
Bacteria (tend)	14			
relative isolation efficiency (%) (t70)	76.2			
Overall assay performance (%) (tend)	88.9			
Expt. id (Date / Name)	KP Chip Trial 3			
Expt. type	Smart centrifugation + Microfluidic detection			
Control plate count	30	28	12	23.3
Expt. plate count	133	134	85	117.3
Volume recovered (ml)	0.5			
Isolation efficiency (%)	99.4			
Concentration in blood (CFU/ml)	52			
Bacteria (t70)	65			
Bacteria (tend)	105			
relative isolation efficiency (%) (t70)	55.4			
Overall assay performance (%) (tend)	89.5			
Expt. id (Date / Name)	2024-02-27 (a)			
Expt. type	Smart centrifugation			
Control plate count	206	210	222	212.7
Expt. plate count	4	2	2	2.7
Volume recovered (ml)	2.7			
Isolation efficiency (%)	120.4			
Concentration in blood (CFU/ml)	17.7			
Expt. id (Date / Name)	2024-02-27 (b)			
Expt. type	Smart centrifugation			
Control plate count	206	210	222	212.7
Expt. plate count	1	1	3	1.7
Volume recovered (ml)	2.7			
Isolation efficiency (%)	75.2			
Concentration in blood (CFU/ml)	17.7			
Expt. id (Date / Name)	2024-02-28 (a)			

Expt. type	Smart centrifugation			
Control plate count	207	234	255	232.0
Expt. plate count	1	0	3	1.3
Volume recovered (ml)	2.5			
Isolation efficiency (%)	67			
Concentration in blood (CFU/ml)	14.7			
Expt. id (Date / Name)	2024-02-28 (b)			
Expt. type	Smart centrifugation			
Control plate count	207	234	255	232.0
Expt. plate count	1	1	4	2.0
Volume recovered (ml)	2.5			
Isolation efficiency (%)	100.6			
Concentration in blood (CFU/ml)	14.7			

SI Table 11: Raw data of plate counts of all experiments with *E. Faecalis.*

				Mean
Expt. id (Date / Name)	2023-09-26			
Expt. type	Smart centrifugation			
Control plate count	7	10	22	13.0
Expt. plate count	7	9	3	6.3
Volume recovered (ml)	2.6			
Isolation efficiency (%)	46.4			
Concentration in blood (CFU/ml)	158			
Expt. id (Date / Name)	2024-02-29 (a)			
Expt. type	Smart centrifugation			
Control plate count	20	26		23
Expt. plate count	1	0	0	0.3
Volume recovered (ml)	2.5			
Isolation efficiency (%)	37.4			
Concentration in blood (CFU/ml)	9.9			
Expt. id (Date / Name)	2024-02-29 (b)			
Expt. type	Smart centrifugation			
Control plate count	20	26		23
Expt. plate count	1	0.0	0.0	0.3
Volume recovered (ml)	2.5			
Isolation efficiency (%)	37.4			
Concentration in blood (CFU/ml)	9.9			
Expt. id (Date / Name)	2023-09-27 (a)			
Expt. type	Smart centrifugation			
Control plate count	94	96	96	95.3
Expt. plate count	99	89		94.0
Volume recovered (ml)	2.7			
Isolation efficiency (%)	97.6			
Concentration in blood (CFU/ml)	1155.6			
Expt. id (Date / Name)	2023-09-27 (b)			
Expt. type	Smart centrifugation			
Control plate count	94	96	96	95.3
Expt. plate count	94	86	78	86.0
Volume recovered (ml)	2.7			

Isolation efficiency (%)	89.3			
Concentration in blood (CFU/ml)	1155.6			
Expt. id (Date / Name)	2023-09-28 (a)			
Expt. type	Smart centrifugation			
Control plate count	101	106	106	104.3
Expt. plate count	7	9	9	8.3
Volume recovered (ml)	2.5			
Isolation efficiency (%)	86.5			
Concentration in blood (CFU/ml)	107			
Expt. id (Date / Name)	2023-09-28 (b)			
Expt. type	Smart centrifugation			
Control plate count	101	106	106	104.3
Expt. plate count	9	9	11	9.7
Volume recovered (ml)	2.5			
Isolation efficiency (%)	100.4			
Concentration in blood (CFU/ml)	107.0			
Expt. id (Date / Name)	2023-09-28 (c)			
Expt. type	Smart centrifugation			
Control plate count	101	106	106	104.3
Expt. plate count	1	1	0	0.7
Volume recovered (ml)	2.6			
Isolation efficiency (%)	60.9			
Concentration in blood (CFU/ml)	12.6			
Expt. id (Date / Name)	2023-09-28 (d)			
Expt. type	Smart centrifugation			
Control plate count	101	106	106	104.3
Expt. plate count	1	0	0	0.3
Volume recovered (ml)	2.6			
Isolation efficiency (%)	30.5			
Concentration in blood (CFU/ml)	12.6			
Expt. id (Date / Name)	2023-09-28 (e)			
Expt. type	Smart centrifugation			
Control plate count	101	106	106	104.3

Expt. plate count	90	66	84	80.0
Volume recovered (ml)	2.5			
Isolation efficiency (%)	70.3			
Concentration in blood (CFU/ml)	1264.6			
Expt. id (Date / Name)	2024-03-01			
Expt. type	Smart centrifugation			
Control plate count	19	21	16	18.7
Expt. plate count	1	0	0	0.3
Volume recovered (ml)	2.6			
Isolation efficiency (%)	94.4			
Concentration in blood (CFU/ml)	4.1			
Expt. id (Date / Name)	2023-10-09			
Expt. type	Smart centrifugation			
Control plate count	15	23	19	19.0
Expt. plate count	1	2	2	1.7
Volume recovered (ml)	2.6			
Isolation efficiency (%)	83.6			
Concentration in blood (CFU/ml)	23			
Expt. id (Date / Name)	2024-02-29 (a)			
Expt. type	Smart centrifugation			
Control plate count	20	26		23.0
Expt. plate count	1	0	0	0.3
Volume recovered (ml)	2.6	2.5		
Isolation efficiency (%)	37.4			
Concentration in blood (CFU/ml)	9.9			
Expt. id (Date / Name)	2024-02-29 (b)			
Expt. type	Smart centrifugation			
Control plate count	20	26		23
Expt. plate count	1	0	0	0.3
Volume recovered (ml)	2.6	2.5		
Isolation efficiency (%)	37.4			
Concentration in blood (CFU/ml)	9.9			
Expt. id (Date / Name)	2024-03-04 (a)			

Expt. type	Smart centrifugation			
Control plate count	35	35	40	36.7
Expt. plate count	0	0	3	1.0
Volume recovered (ml)	2.6			
Isolation efficiency (%)	49.6			
Concentration in blood (CFU/ml)	23.3			
Expt. id (Date / Name)	2024-03-04 (b)			
Expt. type	Smart centrifugation			
Control plate count	35	35	40	36.7
Expt. plate count	1	5	2	2.7
Volume recovered (ml)	2.6			
Isolation efficiency (%)	132.4			
Concentration in blood (CFU/ml)	23.3			
Expt. id (Date / Name)	2024-03-04 (c)			
Expt. type	Smart centrifugation			
Control plate count	35	35	40	36.7
Expt. plate count	0	3	1	1.3
Volume recovered (ml)	2.6			
Isolation efficiency (%)	66.2			
Concentration in blood (CFU/ml)	23.3			
Expt. id (Date / Name)	2024-03-04 (d)			
Expt. type	Smart centrifugation			
Control plate count	35	35	40	36.7
Expt. plate count	0	0	3	1.0
Volume recovered (ml)	2.6			
Isolation efficiency (%)	49.6			
Concentration in blood (CFU/ml)	23.3			
Expt. id (Date / Name)	2024-03-04 (e)			
Expt. type	Smart centrifugation			
Control plate count	324	337		330.5
Expt. plate count	11	13	8	10.7
Volume recovered (ml)	2.6			
Isolation efficiency (%)	58.7			
Concentration in blood (CFU/ml)	209.8			

Expt. id (Date / Name)	2024-03-04 (f)			
Expt. type	Smart centrifugation			
Control plate count	324	337		330.5
Expt. plate count	11	8	9	9.3
Volume recovered (ml)	2.6			
Isolation efficiency (%)	51.4			
Concentration in blood (CFU/ml)	209.8			
Expt. id (Date / Name)	2024-03-04 (g)			
Expt. type	Smart centrifugation			
Control plate count	324	337		330.5
Expt. plate count	5	15	8	9.3
Volume recovered (ml)	2.6			
Isolation efficiency (%)	51.4			
Concentration in blood (CFU/ml)	209.8			
Expt. id (Date / Name)	2024-03-04 (h)			
Expt. type	Smart centrifugation			
Control plate count	324	337		330.5
Expt. plate count	15	13	7	11.7
Volume recovered (ml)	2.6			
Isolation efficiency (%)	64.2			
Concentration in blood (CFU/ml)	209.8			
Expt. id (Date / Name)	2024-03-04 (i)			
Expt. type	Smart centrifugation			
Control plate count	324	337		330.5
Expt. plate count	94	102	75	90.3
Volume recovered (ml)	2.6			
Isolation efficiency (%)	49.7			
Concentration in blood (CFU/ml)	2098.4			
Expt. id (Date / Name)	2024-03-04 (j)			
Expt. type	Smart centrifugation			
Control plate count	324	337		330.5
Expt. plate count	70	88	83	80.3
Volume recovered (ml)	2.6			
Isolation efficiency (%)	44.2			

Concentration in blood (CFU/ml)	2098.4			
Expt. id (Date / Name)	2024-03-04 (k)			
Expt. type	Smart centrifugation			
Control plate count	324	337		330.5
Expt. plate count	94	113	90	99.0
Volume recovered (ml)	2.6			
Isolation efficiency (%)	54.5			
Concentration in blood (CFU/ml)	2098.4			
Expt. id (Date / Name)	2024-03-04 (l)			
Expt. type	Smart centrifugation			
Control plate count	324	337		330.5
Expt. plate count	74	76	100	83.3
Volume recovered (ml)	2.6			
Isolation efficiency (%)	45.9			
Concentration in blood (CFU/ml)	2098.4			
Expt. id (Date / Name)	EF Chip Trial 3			
Expt. type	Smart centrifugation + Microfluidic detection			
Control plate count	167	146	164	159.0
Expt. plate count	158	205	244	202.3
Volume recovered (ml)	0.6			
Isolation efficiency (%)	38.4			
Concentration in blood (CFU/ml)	1012			
Bacteria (t70)	167			
Bacteria (tend)	248			
Overall assay performance (%) (tend)	19.1			
Expt. id (Date / Name)	EF Chip Trial 4			
Expt. type	Smart centrifugation + Microfluidic detection			
Control plate count	23	51	52	42.0
Expt. plate count	303	301	256	286.7
Volume recovered (ml)	0.6			

Isolation efficiency (%)	71.6			
Concentration in blood (CFU/ml)	143			
Bacteria (t70)	10			
Bacteria (tend)	10			
Overall assay performance (%) (tend)	4.3			

References

[1] Anderson, C.M., Pitt, W.G.: Effect of dilution on sedimentational separation of bacteria from blood. Biotechnology Progress **36**(6), 3056 (2020)

[2] Pitt, W.G., Alizadeh, M., Husseini, G.A., McClellan, D.S., Buchanan, C.M., Bledsoe, C.G., Robison, R.A., Blanco, R., Roeder, B.L., Melville, M., *et al.*: Rapid separation of bacteria from blood—review and outlook. Biotechnology progress **32**(4), 823–839 (2016)

[3] Zeng, K., Osaid, M., van der Wijngaart, W.: Efficient filter-in-centrifuge separation of low-concentration bacteria from blood. Lab on a Chip (2023)

[4] Forsyth, B., Torab, P., Lee, J.-H., Malcom, T., Wang, T.-H., Liao, J.C., Yang, S., Kvam, E., Puleo, C., Wong, P.K.: A rapid single-cell antimicrobial susceptibility testing workflow for bloodstream infections. Biosensors **11**(8), 288 (2021)

[5] Narayana Iyengar, S., Kumar, T., Mårtensson, G., Russom, A.: High resolution and rapid separation of bacteria from blood using elasto-inertial microfluidics. Electrophoresis **42**(23), 2538–2551 (2021)

[6] Mach, A.J., Di Carlo, D.: Continuous scalable blood filtration device using inertial microfluidics. Biotechnology and Bioengineering **107**(2), 302–311 (2010). https://doi.org/10.1002/bit.22833

[7] Park, S., Zhang, Y., Wang, T.-H., Yang, S.: Continuous dielectrophoretic bacterial separation and concentration from physiological media of high conductivity. Lab on a Chip **11**(17), 2893–2900 (2011)

[8] Li, S., Ma, F., Bachman, H., Cameron, C.E., Zeng, X., Huang, T.J.: Acoustofluidic bacteria separation. Journal of Micromechanics and Microengineering **27**(1), 015031 (2016)

[9] Ohlsson, P., Evander, M., Petersson, K., Mellhammar, L., Lehmusvuori, A., Karhunen, U., Soikkeli, M., Seppä, T., Tuunainen, E., Spangar, A., von Lode, P., Rantakokko-Jalava, K., Otto, G., Scheding, S., Soukka, T., Wittfooth, S., Laurell, T.: Integrated Acoustic Separation, Enrichment, and Microchip Polymerase Chain Reaction Detection of Bacteria from Blood for Rapid Sepsis Diagnostics. Analytical Chemistry **88**(19), 9403–9411 (2016). https://doi.org/10.1021/acs.analchem.6b00323. Publisher: American Chemical Society. Accessed 2023-01-09

[10] Kim, T.H., Kang, J., Jang, H., Joo, H., Lee, G.Y., Kim, H., Cho, U., Bang, H., Jang, J., Han, S., Kim, D.Y., Lee, C.M., Kang, C.K., Choe,

P.G., Kim, N.J., Oh, M.D., Kim, T.S., Kim, I., Park, W.B., Kwon, S.: Blood culture-free ultra-rapid antimicrobial susceptibility testing. Nature **632**(July 2023) (2024). https://doi.org/10.1038/s41586-024-07725-1

[11] Hallström, E., Kandavalli, V., Wählby, C., Hast, A.: Rapid identification of seven bacterial species using microfluidics, time-lapse phase-contrast microscopy, and deep learning. bioRxiv (2024) https://www.biorxiv.org/content/early/2024/10/17/2024.10.15.618380.full.pdf. https://doi.org/10.1101/2024.10.15.618380

[12] Data and code (2025). Data and code will be distributed by the authors upon request.

[13] Bradski, G.: The opencv library. Dr. Dobb's Journal of Software Tools (2000)

[14] Harris, C.R., Millman, K.J., van der Walt, S.J., Gommers, R., Virtanen, P., Cournapeau, D., Wieser, E., Taylor, J., Berg, S., Smith, N.J., *et al.*: Array programming with NumPy. Nature **585**(7825), 357–362 (2020). https://doi.org/10.1038/s41586-020-2649-2

[15] Virtanen, P., Gommers, R., Oliphant, T.E., Haberland, M., Reddy, T., Cournapeau, D., Burovski, E., Peterson, P., Weckesser, W., Bright, J., *et al.*: Scipy 1.0: fundamental algorithms for scientific computing in python. Nature Methods **17**(3), 261–272 (2020). https://doi.org/10.1038/s41592-019-0686-2

[16] Hunter, J.D.: Matplotlib: A 2d graphics environment. Computing in Science & Engineering **9**(3), 90–95 (2007). https://doi.org/10.1109/MCSE.2007.55

[17] Thevenaz, P., Ruttimann, U.E., Unser, M.: A pyramid approach to subpixel registration based on intensity. IEEE transactions on image processing **7**(1), 27–41 (1998)

[18] Garnier, Simon, Ross, Noam, Rudis, Robert, Camargo, Pedro, A., Sciaini, Marco, Scherer, Cédric: viridis(Lite) - Colorblind-Friendly Color Maps for R. (2024). https://doi.org/10.5281/zenodo.4679423. viridis package version 0.6.5. https://sjmgarnier.github.io/viridis/
